# Enhancing Discovery of Genetic Variants for Posttraumatic Stress Disorder Through Integration of Quantitative Phenotypes and Trauma Exposure Information

**DOI:** 10.1016/j.biopsych.2021.09.020

**Published:** 2021-09-28

**Authors:** Adam X. Maihofer, Karmel W. Choi, Jonathan R.I. Coleman, Nikolaos P. Daskalakis, Christy A. Denckla, Elizabeth Ketema, Rajendra A. Morey, Renato Polimanti, Andrew Ratanatharathorn, Katy Torres, Aliza P. Wingo, Clement C. Zai, Allison E. Aiello, Lynn M. Almli, Ananda B. Amstadter, Soren B. Andersen, Ole A. Andreassen, Paul A. Arbisi, Allison E. Ashley-Koch, S. Bryn Austin, Esmina Avdibegović, Anders D. Borglum, Dragan Babić, Marie Bækvad-Hansen, Dewleen G. Baker, Jean C. Beckham, Laura J. Bierut, Jonathan I. Bisson, Marco P. Boks, Elizabeth A. Bolger, Bekh Bradley, Meghan Brashear, Gerome Breen, Richard A. Bryant, Angela C. Bustamante, Jonas Bybjerg-Grauholm, Joseph R. Calabrese, José M. Caldas-de-Almeida, Chia-Yen Chen, Anders M. Dale, Shareefa Dalvie, Jürgen Deckert, Douglas L. Delahanty, Michelle F. Dennis, Seth G. Disner, Katharina Domschke, Laramie E. Duncan, Alma Džubur Kulenović, Christopher R. Erbes, Alexandra Evans, Lindsay A. Farrer, Norah C. Feeny, Janine D. Flory, David Forbes, Carol E. Franz, Sandro Galea, Melanie E. Garrett, Aarti Gautam, Bizu Gelaye, Joel Gelernter, Elbert Geuze, Charles F. Gillespie, Aferdita Goçi, Scott D. Gordon, Guia Guffanti, Rasha Hammamieh, Michael A. Hauser, Andrew C. Heath, Sian M.J. Hemmings, David Michael Hougaard, Miro Jakovljević, Marti Jett, Eric Otto Johnson, Ian Jones, Tanja Jovanovic, Xue-Jun Qin, Karen-Inge Karstoft, Milissa L. Kaufman, Ronald C. Kessler, Alaptagin Khan, Nathan A. Kimbrel, Anthony P. King, Nastassja Koen, Henry R. Kranzler, William S. Kremen, Bruce R. Lawford, Lauren A.M. Lebois, Catrin Lewis, Israel Liberzon, Sarah D. Linnstaedt, Mark W. Logue, Adriana Lori, Božo Lugonja, Jurjen J. Luykx, Michael J. Lyons, Jessica L. Maples-Keller, Charles Marmar, Nicholas G. Martin, Douglas Maurer, Matig R. Mavissakalian, Alexander McFarlane, Regina E. McGlinchey, Katie A. McLaughlin, Samuel A. McLean, Divya Mehta, Rebecca Mellor, Vasiliki Michopoulos, William Milberg, Mark W. Miller, Charles Phillip Morris, Ole Mors, Preben B. Mortensen, Elliot C. Nelson, Merete Nordentoft, Sonya B. Norman, Meaghan O’Donnell, Holly K. Orcutt, Matthew S. Panizzon, Edward S. Peters, Alan L. Peterson, Matthew Peverill, Robert H. Pietrzak, Melissa A. Polusny, John P. Rice, Victoria B. Risbrough, Andrea L. Roberts, Alex O. Rothbaum, Barbara O. Rothbaum, Peter Roy-Byrne, Kenneth J. Ruggiero, Ariane Rung, Bart P.F. Rutten, Nancy L. Saccone, Sixto E. Sanchez, Dick Schijven, Soraya Seedat, Antonia V. Seligowski, Julia S. Seng, Christina M. Sheerin, Derrick Silove, Alicia K. Smith, Jordan W. Smoller, Scott R. Sponheim, Dan J. Stein, Jennifer S. Stevens, Martin H. Teicher, Wesley K. Thompson, Edward Trapido, Monica Uddin, Robert J. Ursano, Leigh Luella van den Heuvel, Miranda Van Hooff, Eric Vermetten, Christiaan Vinkers, Joanne Voisey, Yunpeng Wang, Zhewu Wang, Thomas Werge, Michelle A. Williams, Douglas E. Williamson, Sherry Winternitz, Christiane Wolf, Erika J. Wolf, Rachel Yehuda, Keith A. Young, Ross McD. Young, Hongyu Zhao, Lori A. Zoellner, Magali Haas, Heather Lasseter, Allison C. Provost, Rany M. Salem, Jonathan Sebat, Richard A. Shaffer, Tianying Wu, Stephan Ripke, Mark J. Daly, Kerry J. Ressler, Karestan C. Koenen, Murray B. Stein, Caroline M. Nievergelt

**Affiliations:** Departments of Psychiatry (AXM, EK, KT, DGB, CEF, WSK, SBN, MSP, VBR, JS, MBS, CMN), Family Medicine and Public Health (AXM, RMS), Radiology (AMD), Neurosciences (AMD), and Cellular and Molecular Medicine (JS), University of California San Diego; Herbert Wertheim School of Public Health and Human Longevity Science (WKT, MBS), University of California San Diego; Moores Cancer Center (TWu), University of California San Diego, La Jolla; Center of Excellence for Stress and Mental Health (AXM, EK, KT, DGB, WSK, VBR, MBS, CMN, SBN), Research Service (EK, KT, VBR, CMN), and Psychiatry Service (DGB), Veterans Affairs Healthcare System; Department of Epidemiology and Health Sciences (RAS), Naval Health Research Center and Division of Epidemiology and Biostatistics (TWu), San Diego State University School of Public Health, San Diego; Department of Psychiatry and Behavioral Sciences (LED), Stanford University, Stanford, California; Departments of Epidemiology (KWC, CAD, ARa, CCZ, BG, MAW, KCK), Social and Behavioral Sciences (SBAu), and Environmental Health (ALR), Harvard T.H. Chan School of Public Health; Psychiatric and Neurodevelopmental Genetics Unit (SR, MJD, KCK), Department of Psychiatry (KWC, JWS), and Analytic and Translational Genetics Unit (JWS), Massachusetts General Hospital; Division of Adolescent and Young Adult Medicine (SBAu), Boston Children’s Hospital; Channing Division of Network Medicine (SBAu), Brigham and Women’s Hospital; Departments of Pediatrics (SBAu), Psychiatry (NPD, EAB, GG, MLK, AK, LAML, AVS, MHT, SW, KJRe), and Health Care Policy (RCK), Harvard Medical School; Biomedical Genetics Section (LAF, MWL, MWM, EJW) and Departments of Neurology (LAF), Ophthalmology (LAF), and Epidemiology (LAF), Boston University School of Medicine; Department of Biostatistics (LAF, MWL), Boston University School of Public Health; Department of Psychological and Brain Sciences (SG) and Dean of Students’ Office (MJL), Boston University; National Center for PTSD (MWL, MWM, EJW), Translational Research Center for TBI and Stress Disorders (REM, WM), and Geriatric Research, Education, and Clinical Center (REM, WM), Veterans Affairs Boston Healthcare System, Boston; Department of Psychology (KAM), Harvard University; Translational Biology (C-YC), Biogen; Stanley Center for Psychiatric Research (NPD, CAD, CCZ, JWS, SR, KCK), Broad Institute of MIT and Harvard; Cohen Veterans Bioscience (MH, HL, ACP), Cambridge; Center of Excellence in Depression and Anxiety Disorders (NPD) and Developmental Biopsychiatry Research Program (MHT), McLean Hospital (EAB, GG, MLK, AK, LAML, AVS, SW, KJRe), Belmont, Massachusetts; Duke Molecular Physiology Institute (RAM, AEA-K, MEG, X-JQ) and Department of Psychiatry and Behavioral Sciences (JCB, MFD, MAH, DEW, NAK), Duke University School of Medicine; Research Service (JCB, MFD, DEW) and Mental Health Service (NAK), Durham Veterans Affairs Medical Center; Genetics Research Laboratory (JCB, MFD, NAK), Veterans Affairs Mid-Atlantic Mental Illness Research, Education, and Clinical Center, Durham; Department of Epidemiology (AEA, TJ), Gillings School of Global Public Health, University of North Carolina at Chapel Hill; Institute for Trauma Recovery (SDL, SAM), Department of Anesthesiology, and Department of Emergency Medicine (SAM), University of North Carolina School of Medicine, University of North Carolina at Chapel Hill, Chapel Hill; GenOmics, Bioinformatics, and Translational Research Center (EOJ) and Fellows Program (EOJ), RTI International, Research Triangle Park, North Carolina; Department of Psychiatry (RP, JG) and National Center for Posttraumatic Stress Disorder (RHP), Veterans Affairs Connecticut Healthcare System, West Haven; Departments of Psychiatry (RP, RHP) and Genetics and Neuroscience (JG), Yale University School of Medicine; Department of Biostatistics (HZ), Yale University, New Haven, Connecticut; Department of Epidemiology (ARa), Columbia University Mailman School of Public Health; Department of Psychiatry (JDF, RY), Icahn School of Medicine at Mount Sinai; Department of Psychiatry (CM, EV), New York University School of Medicine, New York; Department of Mental Health (RY), James J. Peters Veterans Affairs Medical Center, Bronx; Cold Spring Harbor Laboratory (JS), Cold Spring Harbor, New York; Mental Health Services (BB), Division of Mental Health (APW), Atlanta Veterans Affairs Medical Center, Decatur; Departments of Psychiatry and Behavioral Sciences (APW, LMA, BB, CFG, JLM-K, VM, BOR, AKS, JSSt, KJRe) and Gynecology and Obstetrics (AL, AKS), Emory University School of Medicine, Atlanta, Georgia; Department of Psychiatry (ABA, CMS), Virginia Institute for Psychiatric and Behavioral Genetics, Richmond, Virginia; Mental Health Service Line (PAA, CRE, MAP, SRS) and Research Service Line (SGD), Minneapolis Veterans Affairs Health Care System; Department of Psychiatry and Behavioral Sciences (PAA, CRE, MAP, SRS), Medical School, University of Minnesota, Minneapolis, Minnesota; Departments of Psychiatry (LJB, ECN, JPR, NLS) and Genetics (ACH), Washington University in Saint Louis School of Medicine, St. Louis, Missouri; Department of Epidemiology (MB, ESP, ARu, ET), School of Public Health, Louisiana State University Health Sciences Center New Orleans, New Orleans, Louisiana; Division of Pulmonary and Critical Care Medicine (ACB), Department of Internal Medicine, Department of Psychiatry (APK), and Department of Obstetrics and Gynecology (JSSe), University of Michigan Medical School; School of Nursing (JSSe), University of Michigan; Department of Women’s and Gender Studies (JSSe) and Institute for Research on Women and Gender (JSSe), University of Michigan, Ann Arbor, Michigan; Department of Psychiatry (JRC, MRM), University Hospitals Cleveland Medical Center; Department of Psychological Sciences (NCF, AOR), Case Western Reserve University, Cleveland; Department of Psychological Sciences (DLD) and Research and Sponsored Programs (DLD), Kent State University, Kent, Ohio; Center for Military Psychiatry and Neuroscience (AGa, RH), Department of Integrative Systems Biology (MJe), Walter Reed Army Institute of Research, Silver Spring; Department of Psychiatry (RJU), Uniformed Services University, Bethesda, Maryland; Mental Illness Research, Education and Clinical Center (HRK), Corporal Michael J. Crescenz Department of Veterans Affairs Medical Center; Department of Psychiatry (HRK), University of Pennsylvania Perelman School of Medicine, Philadelphia, Pennsylvania; Department of Psychiatry and Behavioral Sciences (IL, KAY), Texas A&M University College of Medicine, Bryan; Research and Development Service (ALP), South Texas Veterans Health Care System; Department of Psychiatry and Behavioral Sciences (ALP), University of Texas Health Science Center at San Antonio, San Antonio; Department of Psychiatry (KAY), Baylor Scott & White Health Central Texas Division, Temple; Center of Excellence for Research on Returning War Veterans (KAY), Central Texas Veterans Health Care System, Waco, Texas; Command (DMa), United States Army, Fort Sill, Oklahoma; Executive Division (SBN), National Center for Post-Traumatic Stress Disorder, White River Junction, Vermont; Department of Psychology (HKO), Northern Illinois University, DeKalb, Illinois; Departments of Psychology (MP) and Psychiatry and Behavioral Sciences (PR-B, LAZ), University of Washington, Seattle, Washington; Departments of Nursing (KJRu) and Psychiatry and Behavioral Sciences (KJRu, ZW), Medical University of South Carolina; Department of Mental Health (ZW), Ralph H. Johnson Veterans Affairs Medical Center, Charleston, South Carolina; Genomics Program (MU), University of South Florida College of Public Health, Tampa, Florida; Social, Genetic and Developmental Psychiatry Centre (JRIC, GB), Institute of Psychiatry, Psychology and Neuroscience, and National Institute of Health Research Maudsley Biomedical Research Centre (JRIC, GB), King’s College London, London; Medical Research Council Centre for Psychiatric Genetics and Genomics (JIB, AE, IJ, CL, BL), National Centre for Mental Health, Cardiff University, Cardiff, United Kingdom; Tanenbaum Centre for Pharmacogenetics (CCZ), Campbell Family Mental Health Research Institute, Centre for Addiction and Mental Health, Molecular Brain Science; Institute of Medical Science (CCZ) and Departments of Laboratory Medicine and Pathology (CCZ) and Psychiatry (CCZ), University of Toronto, Toronto, Ontario, Canada; Research and Knowledge Centre (SBAn, K-IK), The Danish Veteran Centre, Ringsted; Centre for Integrative Sequencing (ADB, PBM), Department of Biomedicine–Human Genetics (ADB), Centre for Integrated Register-Based Research (PBM), and National Centre for Register-Based Research (PBM), Aarhus University; Psychosis Research Unit (OM), Department of Psychiatry, Aarhus University Hospital; The Lundbeck Foundation Initiative for Integrative Psychiatric Research, iPSYCH (ADB, MB-H, JB-G, DMH, OM, PBM, MN, YW, TWe), Aarhus; Department for Congenital Disorders (MB-H, JB-G, DMH), Statens Serum Institut; Departments of Psychology (K-IK) and Clinical Medicine (TWe), University of Copenhagen; Mental Health Center Copenhagen (MN), Mental Health Services in the Capital Region of Denmark, University of Copenhagen; Institute of Biological Psychiatry, Mental Health Services (YW, TWe), Copenhagen University Hospital, Copenhagen; Institute of Biological Psychiatry (WKT), Mental Health Centre Sct. Hans, Roskilde, Denmark; Division of Mental Health and Addiction (OAA), Oslo University Hospital; Norwegian Centre for Mental Disorders Research Centre (OAA), Institute of Clinical Medicine, and Lifespan Changes in Brain and Cognition (YW), Department of Psychology, University of Oslo, Oslo, Norway; Department of Psychiatry (EA), University Clinical Center of Tuzla, Tuzla; Department of Psychiatry (DB), University Clinical Center of Mostar, Mostar; Department of Psychiatry (ADK), University Clinical Center of Sarajevo, Sarajevo, Bosnia and Herzegovina; Departments of Psychiatry (MPB, EG, JJL, DSc) and Translational Neuroscience (JJL, DSc), UMC Utrecht Brain Center, UMC Utrecht; Brain Research and Innovation Centre (EG, EV), Netherlands Ministry of Defence, Utrecht; Department of Psychiatry and Neuropsychology (BPFR), School for Mental Health and Neuroscience, Maastricht University Medical Center, Maastricht; Arq Psychotrauma Research Expert Group (EV), Diemen; Department of Psychiatry (EV), Leiden University Medical Center, Leiden; Departments of Psychiatry (CV) and Anatomy and Neurosciences (CV), VU University Medical Center Amsterdam, Amsterdam, Netherlands; Departments of Psychology (RAB) and Psychiatry (DSi), University of New South Wales, Sydney, New South Wales; Department of Psychiatry (DF, MO) and Phoenix Australia Centre for Posttraumatic Mental Health (MO), University of Melbourne, Melbourne, Victoria; Department of Genetics and Computational Biology (SDG, NGM), QIMR Berghofer Medical Research Institute, Brisbane; School of Biomedical Sciences (BRL, DMe, CPM, JV), Centre for Genomics and Personalised Health (DMe, JV), and School of Psychology and Counseling (RMY), Queensland University of Technology; Jamieson Trauma Institute (CPM, RMY), Metro North Hospital and Health Service, Kelvin Grove; Gallipoli Medical Research Foundation (RM), Green-slopes Private Hospital, Greenslopes, Queensland; Centre for Traumatic Stress Studies (AM, MVH), University of Adelaide, Adelaide, South Australia, Australia; Lisbon Institute of Global Mental Health (JMC-d-A) and Chronic Diseases Research Centre (JMC-d-A), NOVA Medical School, NOVA University of Lisbon, Lisbon, Portugal; South African Medical Research Council Unit on Risk and Resilience in Mental Disorders (SD, DJS, NK), Department of Psychiatry and Neuroscience Institute, and South African Medical Research Council Unit on Child and Adolescent Health (SD), Department of Pediatrics and Child Health, University of Cape Town, Cape Town; South African Medical Research Council/Stellenbosch University Extramural Unit on the Genomics of Brain Disorders (SMJH, SS, LLvdH), Department of Psychiatry (SMJH, SS, LLvdH), Faculty of Medicine and Health Sciences, Stellenbosch University, Stellenbosch, Cape Town, South Africa; Center of Mental Health, Psychiatry, Psychosomatics and Psychotherapy (JD, CW), University Hospital of Würzburg, Würzburg; Department of Psychiatry and Psychotherapy (KD), Faculty of Medicine, University Medical Center Freiburg; Centre for Basics in Neuromodulation (KD), Faculty of Medicine, University of Freiburg, Freiburg; Department of Psychiatry and Psychotherapy (SR), Charité – Universitätsmedizin, Berlin, Germany; Department of Psychiatry (AGo), University Clinical Center of Kosovo, Pristina, Kosovo; Department of Psychiatry (MJa), University Hospital Centre Zagreb, Zagreb, Croatia; and Department of Medicine (SES), Universidad Peruana de Ciencias Aplicadas Facultad de Ciencias de la Salud, Lima, Peru.

## Abstract

**BACKGROUND:**

Posttraumatic stress disorder (PTSD) is heritable and a potential consequence of exposure to traumatic stress. Evidence suggests that a quantitative approach to PTSD phenotype measurement and incorporation of lifetime trauma exposure (LTE) information could enhance the discovery power of PTSD genome-wide association studies (GWASs).

**METHODS:**

A GWAS on PTSD symptoms was performed in 51 cohorts followed by a fixed-effects meta-analysis (*N* = 182,199 European ancestry participants). A GWAS of LTE burden was performed in the UK Biobank cohort (*N* = 132,988). Genetic correlations were evaluated with linkage disequilibrium score regression. Multivariate analysis was performed using Multi-Trait Analysis of GWAS. Functional mapping and annotation of leading loci was performed with FUMA. Replication was evaluated using the Million Veteran Program GWAS of PTSD total symptoms.

**RESULTS:**

GWASs of PTSD symptoms and LTE burden identified 5 and 6 independent genome-wide significant loci, respectively. There was a 72% genetic correlation between PTSD and LTE. PTSD and LTE showed largely similar patterns of genetic correlation with other traits, albeit with some distinctions. Adjusting PTSD for LTE reduced PTSD heritability by 31%. Multivariate analysis of PTSD and LTE increased the effective sample size of the PTSD GWAS by 20% and identified 4 additional loci. Four of these 9 PTSD loci were independently replicated in the Million Veteran Program.

**CONCLUSIONS:**

Through using a quantitative trait measure of PTSD, we identified novel risk loci not previously identified using prior case-control analyses. PTSD and LTE have a high genetic overlap that can be leveraged to increase discovery power through multivariate methods.

Posttraumatic stress disorder (PTSD) may develop after exposure to traumatic life events. PTSD can severely impact the mental and physical health of affected individuals and impair their interpersonal relationships (1). While the estimated community prevalence of PTSD in the United States is 5% to 10% (2), the rate of PTSD differs based on the nature of trauma exposure (3) and other environmental (4) and genetic (5–7) factors. Identifying the biological mechanisms associated with the etiology of PTSD will facilitate the discovery of biomarkers for screening and diagnostic purposes (7) and the development of new treatments.

Genome-wide association studies (GWASs) facilitate biological understanding of PTSD (8,9), but are well known to be limited by statistical power to identify risk variation (10). Quantitative measures of PTSD enhance discovery power over binary trait definitions (9,11). Appropriately accounting for trauma exposure hypothetically enhances power, as individuals will not develop the disorder unless they are exposed to trauma, regardless of high genetic vulnerability for PTSD (12,13). Moreover, the notion that genetic variants can pre-dispose to trauma exposure is only starting to be explored (14). As trauma exposure is a prerequisite for the development and manifestation of PTSD, investigating the genetics of trauma exposure will hypothetically lead to a clearer picture of PTSD genetics.

The Psychiatric Genomics Consortium (PGC)–PTSD is a global collaborative effort to study the genetic basis of PTSD through meta-analysis of diverse cohorts (13). Subsequent to a case-control GWAS (8), our collaborators have provided quantitative measures of PTSD and lifetime trauma exposure (LTE). To obtain genomic insights from the quantitative PTSD phenotyping, we performed a GWAS of PTSD symptoms in 182,199 participants from the PGC-PTSD Freeze 2 dataset. To determine if accounting for LTE would provide the hypothesized increase in discovery power, we performed a GWAS of PTSD with covariate adjustment for LTE, showing that it lowers PTSD signal. We investigated the possibility that multicollinearity arising from high genetic correlation (*r*_*g*_) of PTSD and LTE was responsible for this result. To perform this investigation, we performed a GWAS of LTE in the most powered and unbiased (15) subsample of the data, 132,988 participants from the UK Biobank (UKBB) (16), then evaluated the *r*_*g*_ of PTSD and LTE. To explore the *r*_*g*_ further, we contrasted the *r*_*g*_s that PTSD and LTE have with other traits. We showed that the high *r*_*g*_ of PTSD and LTE can be leveraged to enhance the power of PTSD GWASs using multivariate methods. We replicated PTSD GWAS findings in the Million Veteran Program (MVP) GWAS of total PTSD symptoms (MVP_TOT_). We contextualized genomic findings through functional annotation, tissue expression analyses, and phenome-wide association study (PheWAS).

## METHODS AND MATERIALS

### Study Population and Phenotyping

Participants were drawn from a collection of 51 cohorts within the PGC-PTSD Freeze 2 dataset, as previously described in Nievergelt *et al*. (8). All participants included in the present study were of genetically estimated European ancestry. PTSD symptoms and LTE were measured within each cohort using structured clinical interviews, self-reported inventories, or clinical evaluation. A summary of the assessment and scoring methods for the various studies is presented in Table S1 in Supplement 2, and a complete description is available in Nievergelt *et al*. (8). All participants provided written informed consent, and studies were approved by the relevant institutional review boards and the University of California San Diego Human Research Protection Program.

### GWAS Quality Control

Genotyping, quality control (QC), and imputation methods for the included studies have been described in detail (8). In brief, participating cohorts provided phenotype and genotype data or GWAS summary statistics to the PGC-PTSD for quality control and analysis. For studies in which the PGC-PTSD analyst had direct access to genotype data, RICOPILI (17) was used to perform QC and imputation. QC included standard filters for single nucleotide polymorphism (SNP) call rates (exclusion of SNPs with call rate <98% or a missing difference >0.02 between cases and controls), call rate for participant genotypes (samples with <98% call rate excluded), Hardy-Weinberg equilibrium (*p* < 1 × 10^−6^ in controls), and heterozygosity (within ± 0.2). Datasets were phased using SHAPEIT (18) and imputed using IMPUTE2 (19) with the 1000 Genomes Phase 3 reference panel data (20). For the UKBB, QC and imputation were carried out centrally by UKBB investigators as previous described (16) and GWAS was carried out by the PGC-PTSD analyst. For cohorts with data-sharing restrictions, analyses were performed using similar protocols by the study team that had individual-level data access, and GWAS summary statistics were provided to the PGC-PTSD.

### Genome-wide Association Study

Only unrelated (π < 0.2) participants were retained for analysis. Principal components (PCs) were calculated within each cohort using EIGENSOFT v6.3.4 (21). The PTSD GWAS was performed within cohorts using PLINK 2.0 alpha with the −glm option, with the exception of UKBB and VETSA (Vietnam Era Twin Study of Aging) data, which were analyzed using BOLT-LMM v2.3.4 (22). Where available, PTSD symptom scores were analyzed using linear regression (*n* = 36 cohorts); PTSD case-control status was used if symptom scores were not available, using logistic regression (*n* = 15 cohorts). In both cases, 5 PCs were included as covariates to account for population stratification and genotyping artifacts. The UKBB PTSD GWAS included an additional PC as well as batch and assessment center covariates. Studies providing summary data used similar analytic strategies, as previously described (8). For each GWAS, SNPs with minor allele frequency <1% or imputation information score <0.6 were excluded. To perform a GWAS of PTSD conditioned on LTE, the GWAS was performed with LTE included as an additional covariate as either a count of LTEs or a binary variable, depending on data availability. The GWAS of the LTE count phenotype in the UKBB sample was performed in BOLT-LMM using 6 PCs, batch, and assessment center as covariates.

### PTSD Meta-analysis

Sample size–weighted fixed-effects meta-analysis was performed using METAL (23). To account for different analytic methods and measure scales, effect estimates were converted into *z* scores by dividing effect sizes by standard errors (24). Case-control and quantitative GWAS subsets were evaluated for *r*_*g*_ to determine if they could be meta-analyzed. To account for differences in ascertainment, heritability, and power between case-control and quantitative subsets, modified sample size weights were derived as previously described (25), assuming 10% population prevalence of PTSD, the estimates of SNP-based heritability (*h*^2^_SNP_), *r*_*g*_, and sample PTSD prevalence. Meta-analysis was conducted on the reweighted *z* scores. Only SNPs available in >90% of all samples (*N* ≥ 163,979) were included in analyses. Regional annotation plots of genome-wide significant loci were produced using Locus-Zoom (26).

### Heritability and Genetic Correlation Estimation With Linkage Disequilibrium Score Regression

Trait *h*^2^_SNP_ and *r*_*g*_ were estimated from GWAS summary statistics using linkage disequilibrium score regression (27). The linkage disequilibrium score intercept was used to test for inflation of test statistics owing to residual population stratification or other artifacts, and the attenuation factor {[intercept − 1]/[mean (χ^2^) − 1]} was used to determine the proportion of inflation of test statistics owing to residual population stratification (Table S2 in Supplement 2). Heritabilities were contrasted using a *z* test where standard errors were estimated using the block-jackknife approach. To estimate *r*_*g*_ with other disorders, the LD Hub web interface was used (28). To identify genetic differences between PTSD and LTE, the *r*_*g*_s observed for PTSD and LTE were contrasted using *z* tests, where significance level was determined using Bonferroni correction for the 772 traits tested (*p* < 6.47 × 10^−5^).

### FUMA

FUMA v1.3.6a (29) was used with the default settings (Supplement 1) to visualize and annotate GWAS results. The FUMA pipeline integrates the MAGMA (30) tool to perform gene-based, gene-pathway, and tissue-enrichment analyses, with significance based on Bonferroni correction. 1000 Genomes Europeans were used as reference genotypes. Tissue-enrichment analysis included Genotype-Tissue Expression (GTEx) v8 expression data (31).

### *Cis*-Quantitative Trait Locus Mapping

The effects of GWAS loci on transcriptomic regulation of surrounding genes (locus within ± 1 Mb of the gene transcription starting site) were tested for 49 tissues in GTEx v8 with genome-wide false discovery rate correction applied. Using the same criteria, GTEx v8 data were also used to investigate the effects of GWAS loci on the regulation of alternative splicing isoforms. A detailed description regarding GTEx v8 quantitative trait locus (QTL) mapping data by the GTEx Consortium is available (32). Briefly, *cis*-expression QTL (eQTL) and *cis*-splicing QTL mapping was performed using FastQTL (33) including the top 5 genotyping PCs, probabilistic estimation of expression residuals factors (34), sequencing platform, sequencing protocol, and sex as covariates.

### Replication Analysis

Summary data from MVP_TOT_ (dbGaP study accession phs001672.v4.p1) was used to replicate GWAS results. MVP_TOT_ included 186,689 European ancestry participants who completed the PTSD Checklist–Civilian Version and passed QC. Details of MVP_TOT_ have been published (35). SNPs were deemed replicated in MVP_TOT_ if they had matching effect direction and were nominally significant after Bonferroni correction for the 9 SNPs tested (*p* < .006).

### Multi-Trait Analysis of GWAS

Multi-Trait Analysis of GWAS (MTAG) (36) performs multivariate analysis of genetically correlated traits to increase discovery power for each input trait, providing trait-specific effect estimates and *p* values. MTAG was used to perform multivariate analysis with PTSD and LTE GWASs. The maxFDR statistic was used to test for MTAG model assumptions (Supplement 1).

### Phenome-wide Association Study

To understand further how functional changes of significant loci are associated with human traits and diseases, we conducted a PheWAS of leading SNPs from PTSD and LTE loci using data from the GWAS Atlas (available at https://atlas.ctglab.nl/) (37). Bonferroni correction was applied to account for the 4756 phenotypes available that were tested (*p* < 1.05 × 10^−5^).

## RESULTS

The PTSD GWAS meta-analysis included 182,199 participants of European ancestry from 51 cohorts (Table S1 in Supplement 2). The largest cohort was the UKBB (*N* = 134,586 participants). Across the cohorts, PTSD was assessed using a variety of different methods (*n* = 19 methods); the most common methods were versions of the Clinician-Administered PTSD Scale (*n* = 18 studies) and PTSD Checklist (*n* = 14 studies). The majority of participants (91%, *n* = 165,825, 36 studies) were analyzed based on PTSD symptom scores; the remaining participants (9%, *n* = 16,374, 15 studies) did not have symptom scores available and were analyzed based on PTSD case-control status.

### PGC-PTSD GWAS Meta-analysis

**Figure 1.**
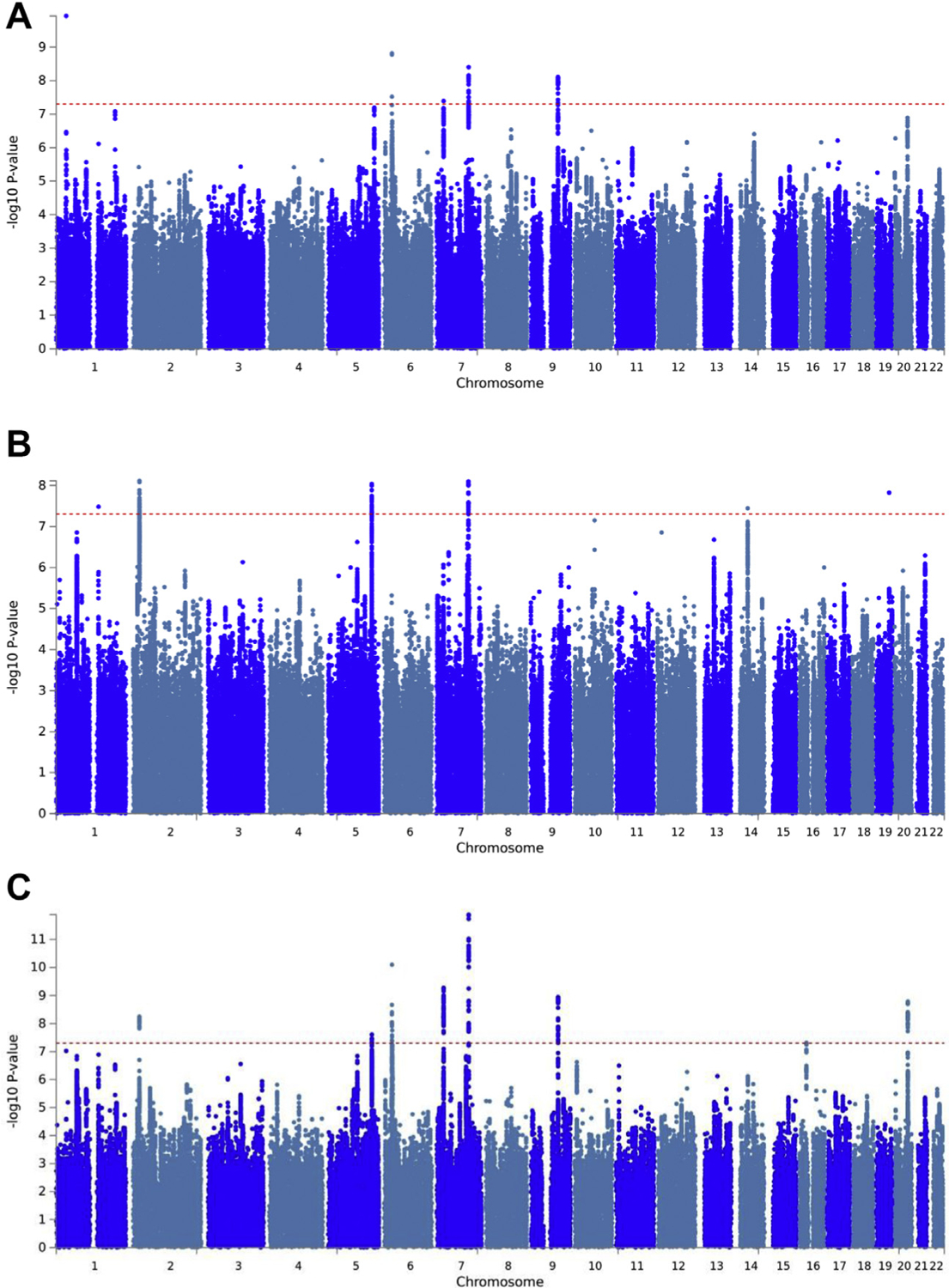
Manhattan plots of genome-wide association study (GWAS) associations. The x-axis is the position on the genome, ordered by chromosome and base-pair position. The y-axis is the −log_10_ *p* value of association. Each dot represents the association between a given single nucleotide polymorphism and the trait. Colors alternate between chromosomes, with odd chromosomes colored blue and even chromosomes colored teal. **(A)** Results of posttraumatic stress disorder GWASs. **(B)** Results of lifetime trauma exposure GWASs. **(C)** Posttraumatic stress disorder–specific results of MTAG (Multi-Trait Analysis of GWAS) analysis of posttraumatic stress disorder and lifetime trauma exposure.

The *h*^2^_SNP_ of meta-analysis of cohorts analyzed by symptom scores was 0.0547 (SE = 0.0042, *p* = 8.9 × 10^−39^) (Table S2 in Supplement 2). The *h*^2^_SNP_ was similar, albeit not significant, in the smaller meta-analysis of case-control cohorts (observed scale *h*^2^_SNP_ = 0.0580, SE = 0.0259, *p* = .17). The *r*_*g*_ between the symptom score and case-control analyses was very high (*r*_*g*_ = 0.9646, SE = 0.36, *p* = .0074). Thus, symptom score and case-control GWASs were meta-analyzed. We identified 5 genome-wide significant loci (Table 1, Figure 1A). Leading variants in significant loci mapped to an intergenic locus on chromosome 1, the intronic region of the *GABBR1* gene on chromosome 6, the intronic regions of *MPP6* and *DFNA5* on chromosome 7, an intron of *FOXP2* on chromosome 7, and the intronic region of *FAM120A* on chromosome 9. Gene-based analysis identified 6 significant genes (*DCAF5, EXD2, FAM120A, FOXP2, GALNT16*, and *PHF2*) (Table S3 in Supplement 2).

### PGC-PTSD GWAS Covariate Adjustment for LTE

We repeated the GWAS of PTSD with covariate adjustment for LTE. *h*^2^_SNP_ was 0.0389 (SE = 0.00340, *p* = 2.6 × 10^−30^), 31% lower than the PTSD GWAS without LTE covariate adjustment (*p* = 8.6 × 10^−20^). There was a genome-wide significant locus in an uncharacterized region, *CTC-340A15.2*, on chromosome 5 that was not identified in the PTSD GWAS (Table S4 in Supplement 2). Effects changed slightly for the loci previously identified in the unadjusted PTSD GWAS (Table S4 in Supplement 2). Gene-based analysis identified no significant genes.

### UKBB LTE GWAS

We performed GWAS of LTE count in the UKBB subset of the PGC-PTSD GWAS data (132,988 UKBB participants). Of participants, 30.9% reported 1 LTE, 14.8% reported 2 LTEs, 6.3% reported 3 LTEs, and 3.3% reported 4 or more LTEs (Table S5 in Supplement 2). The *h*^2^_SNP_ of LTE count was 0.0734 (SE = 0.005, *p* = 8.7 × 10^−49^). Six loci showed genome-wide significance (Figure 1B, Table 2). Leading variants in significant loci mapped to an intron of *PRUNE* on chromosome 1, the intron of noncoding RNA AC068490.2 on chromosome 2, the intron of *SGCD* on chromosome 5, an intron of *FOXP2* on chromosome 7 (also identified in the PGC-PTSD GWAS), an intergenic region in chromosome 14 near *MDGA*, and upstream of *CCDC8* on chromosome 19. Gene-based analysis identified *SGCD* (chromosome 5: 155,297,354–156,194,799 base pairs, 2965 SNPs, 99 parameters, *z* = 5.53, *p* = 1.5 × 10^−8^) and *C20orf112* (chromosome 20:31,030,862–31,172,876 base pairs, 296 SNPs, 21 parameters, *z* = 4.73, *p* = 1.13 × 10^−6^). GWAS of LTE count weighted by trauma-specific PTSD prevalences yielded highly similar results, being highly genetically correlated to the unweighted count (*r*_*g*_ = 1, SE = 0.0016, *p* < 1.13 × 10^−100^).

### Genetic Overlap Between LTE and PTSD

**Figure 2.**
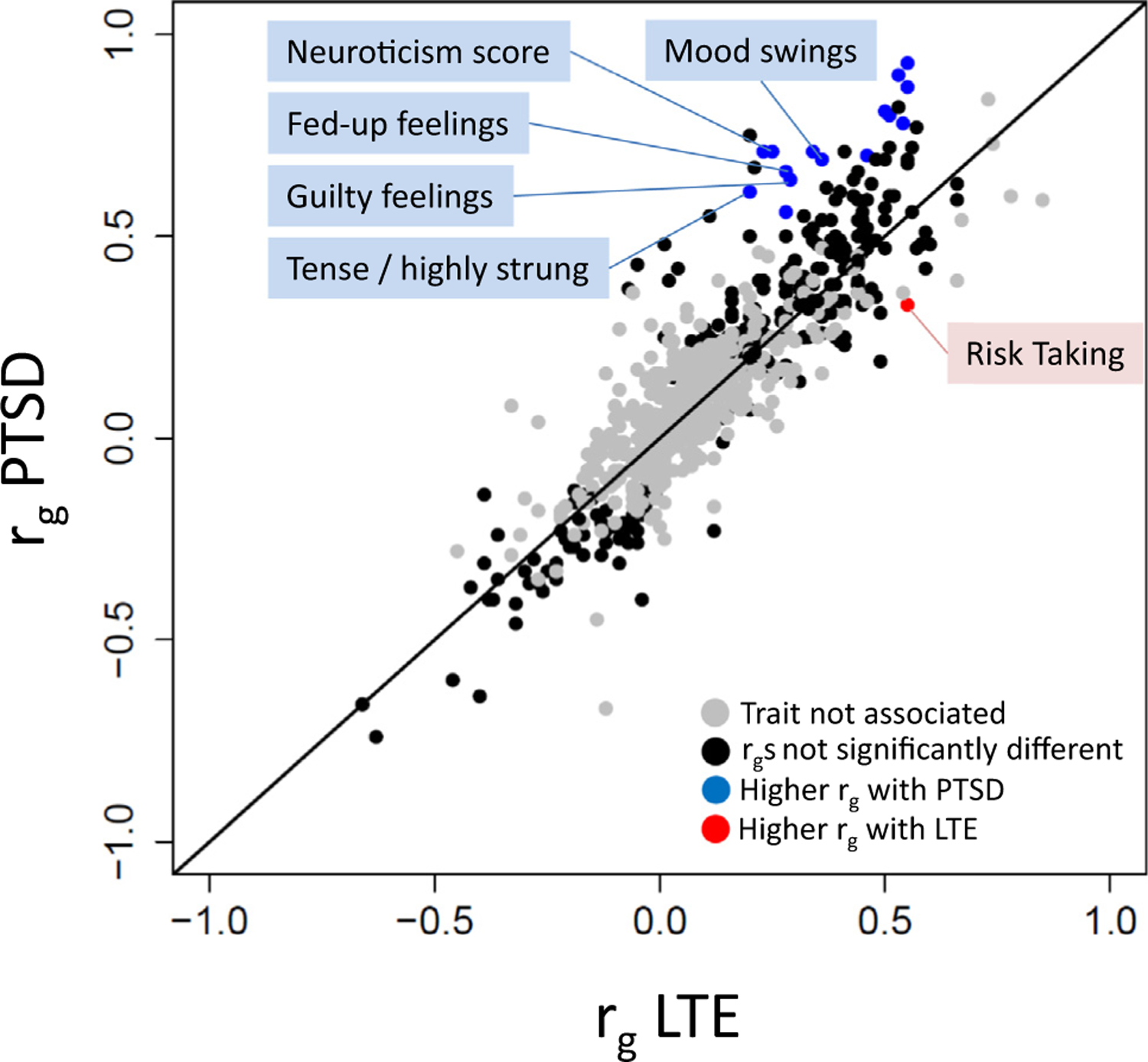
Comparison of the genetic correlations of posttraumatic stress disorder (PTSD) and lifetime trauma exposure (LTE) with other traits. The x-axis is the genetic correlation between LTE and a given trait from the LD Hub. The y-axis is the genetic correlation between PTSD and a given trait. Each dot depicts a given trait. Colored (black, red, or blue) dots indicate traits with significant genetic correlation to both PTSD and LTE after Bonferroni adjustment. Noncolored (gray) dots indicate traits where genetic correlation is not significant after Bonferroni adjustment. Blue dots indicate traits with significantly higher genetic correlation with PTSD than with LTE. Red dots indicate traits with significantly higher correlation with LTE than with PTSD. The top 5 traits with a significantly higher correlation to PTSD than LTE and top trait with significantly higher correlation to LTE than PTSD have been labeled.

The *r*_*g*_ between PTSD and LTE was high (*r*_*g*_ = 0.7239, *p* < 1 × 10^−100^). To explore this genetic overlap, we contrasted patterns of *r*_*g*_ of PTSD and LTE to other traits. Testing 772 human traits and diseases, we observed 269 and 217 *r*_*g*_s that survived Bonferroni multiple testing correction (*p* < 6.47 × 10^−5^) for PTSD and LTE, respectively (Table S6 in Supplement 2). There was complete directional concordance between PTSD and LTE among the 187 *r*_*g*_s that were significant in both analyses. For several traits, while the effect direction was concordant, the magnitude of correlation with PTSD was significantly different from the correlation with LTE (*p* < 6.47 × 10^−5^) (Figure 2). Fifteen traits showed significantly higher genetic correlation with PTSD than with LTE (e.g., neuroticism score *p* = 2.74 × 10^−24^; fed-up feelings *p* = 1.83 × 10^−15^; mood swings *p* = 9.92 × 10^−15^; loneliness *p* = 8.07 × 10^−8^; depressive symptoms *p* = 1.94 × 10^−7^; irritability *p* = 2.27 × 10^−7^). Conversely, risk taking showed a significantly higher genetic correlation with LTE (*r*_*g*_ = 0.55, *p* = 2.71 × 10^−55^) than with PTSD (*r*_*g*_ = 0.33, *p* = 3.9 × 10^−20^; *p* = 8.09 × 10^−6^).

### Multivariate Analysis of PTSD and Trauma Exposure

MTAG analysis that combined PTSD GWAS meta-analysis and UKBB LTE GWAS reported an effective sample size increase of PTSD GWAS from 182,199 to 217,491. There were 8 genome-wide significant loci for the MTAG PTSD analysis, including 4 loci not identified in the PTSD GWAS meta-analysis (Table 1, Figure 1C). Leading variants from additional loci mapped to an intergenic region in chromosome 2, the intron of *SGCD* on chromosome 5, an intergenic region on chromosome 16 near *ZKSCAN2* and *AQP8*, and the intron of *STAU1* on chromosome 20. In gene-based analysis, there were 8 significant genes, including 5 genes not identified from the original GWAS gene-based analysis (*CSE1L, DFNA5, FOXP1, SGCD, TRIM26*) (Table S3 in Supplement 2).

### Cross-Replication in MVP_TOT_

Of the 9 loci identified across the PTSD GWASs (5 from the PGC GWAS and 4 from the MTAG), 4 replicated significantly in MVP_TOT_ (*p* < .006) (Table 1, Figures S2–S10 in Supplement 1). Of the 11 genes identified in gene-based analyses (6 in the GWAS and 5 in the MTAG), 7 replicated at least at a nominally significant level in MVP_TOT_ (Table S3 in Supplement 2). Additionally, of 15 loci identified in MVP_TOT_ GWASs, 9 nominally replicated in PGC-PTSD (Table S7 in Supplement 2). Overall, *r*_*g*_ between PGC-PTSD and MVP_TOT_ was high (*r*_*g*_ = 0.8359, SE = 0.0376, *p* = 2.5 × 10^−109^).

### Functional Consequences of Risk Loci

We examined the functional impact of the 9 GWAS variants associated with PTSD (5 from the GWAS and 4 from the MTAG) (Table 1). We observed that 7 loci were related to multiple tissue-specific eQTLs (Table S8 in Supplement 2), where 11% of false discovery rate–significant eQTLs were in brain regions. A similar trend was present for splicing QTLs (Table S9 in Supplement 2), where only 7% of gene-tissue combinations were related to brain regions. Further details of the eQTL analysis are provided in Supplement 1.

We found enrichment of genes involved in brain transcriptomic regulation in PTSD (Table S10 in Supplement 2). All brain regions tested were at least nominally significant, with several remaining significant after Bonferroni correction (MTAG: cortex *p* = 2.9 × 10^−5^, frontal cortex Brodmann area (BA) 9 *p* = 3.53 × 10^−5^, cerebellum *p* = 1.09 × 10^−4^, anterior cingulate cortex BA 24 *p* = 1.29 × 10^−4^, cerebellar hemisphere *p* = 1.43 × 10^−3^, nucleus accumbens/basal ganglia *p* = 3.6 × 10^−4^). There was no significant enrichment detected in any sets from the list of curated gene sets and Gene Ontology terms (Table S11 in Supplement 2).

### Phenome-wide Association Study

We identified 200 phenome-wide significant associations (Table S12 in Supplement 2), with more than half of the significant associations related to two domains: psychiatry (34%) and metabolism (18%). The strongest PheWAS associations with PTSD and LTE loci included height and body mass phenotypes, educational attainment, social interaction, sexual activity, risk tolerance, and sleep phenotypes (Supplement 1). Several PTSD loci showed widespread pleiotropy across multiple psychiatric traits: rs10266297 (35 significant associations, 40% psychiatric domain, top psychiatric result: risk taking *p* = 1.27 × 10^−11^), rs10821140 (37 significant associations, 38% psychiatric domain, top psychiatric result: loneliness *p* = 1.11 × 10^−11^), rs146918648 (44 significant associations, 48% psychiatric domain, top psychiatric result: tenseness/restlessness *p* = 2.13 × 10^−9^).

## DISCUSSION

Our GWASs aimed to advance understanding of PTSD genetics by integrating quantitative PTSD phenotypes and LTE exposure information in 182,199 participants of European ancestry from 51 cohorts. Overall, quantitative PTSD phenotyping captured similar genetic signal to our prior case-control analysis (*r*_*g*_ = 0.92–1.14) (8), but with substantially higher power. However, by using LTE as a covariate, which hypothetically accounts for unexpressed genetic vulnerability among unexposed participants (12), we found a significant reduction in heritability and gene discovery. As high *r*_*g*_ between PTSD and LTE would be one hypothetical explanation for this result (i.e., multicollinearity), we performed a GWAS of LTE and contrasted it to GWAS results for PTSD. We found that LTE has *h*^2^_SNP_ comparable to PTSD and high *r*_*g*_ compared with PTSD. We leveraged the *r*_*g*_ to significantly enhance PTSD discovery power using a multivariate approach (36).

One explanation for *h*^2^_SNP_ of PTSD adjusted for LTE being lower than the unadjusted estimate is that it may have removed genetic effects on PTSD mediated by trauma exposure (12,13). Given that trauma is a prerequisite for PTSD, genetic effects on trauma exposure can have mediated (i.e., indirect) effects on PTSD. Indeed, this seems plausible, as our LTE GWAS suggested a substantial amount of *h*^2^_SNP_ related to trauma exposure. Therefore, the estimated *h*^2^_SNP_ of PTSD conditional on LTE would theoretically reflect only nonmediated (i.e., direct) effects and thus would be smaller.

We used *r*_*g*_ to quantify the genetic overlap between LTE and PTSD, finding similar magnitude to findings from twin studies (5,6). At the same time, incomplete *r*_*g*_ between these two phenotypes also suggested meaningful genetic differences. To investigate this, we contrasted the magnitudes of *r*_*g*_ that PTSD and LTE shared with other traits. For most traits, *r*_*g*_ with PTSD was quite similar in magnitude to *r*_*g*_ with LTE. However, we also found that negative affect traits, such as neuroticism and irritability, were more strongly correlated with PTSD than LTE, whereas risk-taking behavior showed higher correlation with LTE than PTSD. This suggests that some variants influence PTSD and LTE through somewhat distinct psychological and behavioral mechanisms (5).

The high *r*_*g*_ between PTSD and LTE facilitates the application of multivariate approaches to PTSD GWASs. Whereas the *r*_*g*_ between PTSD and LTE induces loss of power in the PTSD analysis when conditioned on LTE, a multivariate approach can benefit from it. Our multivariate (36) analysis resulted in a 19% increase in the effective sample size by adding LTE count data from the UKBB and identified replicable loci and patterns of tissue expression not identified in a standard PTSD GWAS.

The biological mechanisms associated with several of the protein products of identified genes have been linked to PTSD pathophysiology in animal and cell models: amygdala-mediated fear extinction [FAM120A (38)], neuronal transcriptional regulation [FOXP2 (39)], brain excitatory/inhibitory balance [ARFGEF2, GABBR1, STAUI1 (40)], intracellular vesicular trafficking and other synaptic activities [ARFGEF2 (41), MPP6 (42), SEMA6C (43), SGCD (44)], and inflammation [HIATL1, TRIM26 (45), TRIM27 (46), ZMYM4, ZNF165 (47)]. Blood and brain transcription-wide association and differential gene expression studies of PTSD have also implicated some of these genes, including a blood-based prediction of downregulation of *ARFGEF2* in the dorsolateral prefrontal cortex (48) and a postmortem study of human PTSD cortex indicating downregulation of CTSS expression in the dorsal anterior cingulate cortex and downregulation of *OSBPL3* expression in the dorsolateral prefrontal cortex (49).

Interestingly, PTSD loci show widespread pleiotropic associations in PheWAS (50–52). Some loci point to factors associated with existing clinical presentations of PTSD (e.g., sleep), while others point to potential risk/protective factors for PTSD, such as educational attainment and cognitive functioning. Loci may affect PTSD through their direct influence on these risk/protective factors. Alternatively, the high degree of pleiotropy shown by these loci suggests that they could influence PTSD risk through a more general alteration of biological function (37), such as general predisposition to psychiatric illness (53). In particular, metabolic phenotypes such as height and body mass also appeared to be enriched in our PheWAS. This could be the influence of these loci on previously implicated inflammatory mechanisms for PTSD (8) or simply an artifact of their overrepresentation in the GWAS Atlas. Nevertheless, the broad variety of behavioral and clinical domains associated with these loci suggest complex etiologic heterogeneity of PTSD that could relate to subtypes (54).

Further characterization of significant loci via eQTL analyses identified expression across a variety of tissue types. Given the high degree of shared eQTL architecture between tissues, the presence of some of these tissues might not be directly related to PTSD pathogenesis. Indeed, on the genome-wide level, our tissue enrichment analysis suggests that only brain tissues are relevant. The brain regions implicated are consistent with functional magnetic resonance imaging and structural magnetic resonance imaging findings of PTSD. BA 24 (as part of the ventral anterior cingulate cortex) is implicated in PTSD response to trauma-, fear-, and threat-related stimuli (55,56). BA 9 (as part of the dorsomedial prefrontal cortex) reflects response to self-referential thought, theory of mind, empathy, and moral judgments and shows greater engagement in people with PTSD and trauma-exposed individuals (55,57,58). Nucleus accumbens expression is consistent with the neuroimaging evidence of its role in the reward system, which is prominently affected with emotional numbing symptoms of PTSD (59–62).

### Limitations

Stress-related disorders are phenotypically complex and heterogeneous (63), which limits discovery power and complicates translation to clinical application. The strategies proposed for understanding and addressing heterogeneity in major depressive disorder, such as harmonization of measures, additional phenotypic measures, and investigations of subtypes, could be applied to PTSD as additional avenues to enhance discovery power (64). Sex differences may also contribute a significant source of heterogeneity (8,65–68). Our analyses were restricted to participants of European ancestry given power limitations for other ancestry groups. However, urgent scientific and ethical reasons call for extending analyses to individuals of non-European ancestry (69). The PGC-PTSD group has actively been gathering data to increase representation from diverse ancestry and developing methods to optimize analyses in admixed populations (70). As sample sizes increase, future investigations will be powered to investigate ancestry and sex-specific genetic influences on PTSD and trauma exposure. In performing a GWAS of cumulative LTE, we identified several significant loci, including loci previously identified in GWASs of childhood trauma exposure (14). A full investigation of the genetic basis of LTE is clearly warranted. Future work could also examine the relationship between PTSD and specific types or numbers of trauma exposure, as they plausibly have different relationships with PTSD (6) and may therefore be more informative than our cumulative measure for LTE. Finally, trauma was assessed via participant self-report, which may vary with mood and PTSD symptoms at the time of reporting (71) and could inflate genetic associations with PTSD.

## Conclusions

Novel replicable risk loci for PTSD identified by incorporating quantitative symptom data and trauma exposure information into GWASs offer us new insights into the genetic architecture of PTSD. Beyond the nature of LTE as an environmental exposure, there is a heritable component to LTE that overlaps highly with PTSD to impart an enhanced understanding of PTSD genetics. In future investigations, the genetic architectures of PTSD and LTE could be further delineated using causal mediation analysis (72), which can provide estimates of LTE-related mediation and gene-by-environment interaction. Our results reinforce the notion that in addition to larger samples, more detailed phenotyping and sophisticated modeling are needed to account for the role of environmental exposure in developing PTSD, as these influence GWAS discovery power. Widespread pleiotropy of significant loci suggests that cross-disorder analysis with PTSD (73,74) will enhance our understanding of how these loci modify risk for PTSD and related disorders.

## Supplementary Material

mmc2

mmc3

## Figures and Tables

**Table 1.** Genome-wide Significant Loci From PTSD GWASs and MTAGs With Replication in MVP_TOT_ GWAS

						PGC-PTSD GWAS			PGC-PTSD MTAG		MVP_TOT_		
Analysis	rsID	Chr	Position^a^	A1	A2	A1 Freq	*z* Score	*p* Value	*z*	*p* Value	A1 freq	*z* Score	*p* Value^b^
Identified in GWAS	rs72657988	1	35688541	T	G	0.08	6.44	1.2 × 10^−10^	5.34	9.4 × 10^−8^	0.07	2.18	.029
	rs146918648	6	28548674	A	G	0.04	6.04	1.5 × 10^−9^	6.50	8.0 × 10^−11^	0.04	2.00	.045
	rs2721816^c^	7	24699329	A	G	0.82	−5.27	1.4 × 10^−7^	−5.80	6.5 × 10^−9^	0.82	−1.45	.15
	rs10266297	7	114143407	T	C	0.59	5.38	7.4 × 10^−8^	6.72	1.8 × 10^−11^	0.59	4.97	6.7 × 10^−7^
	rs10821140	9	96253169	A	C	0.35	−5.71	1.2 × 10^−8^	−6.02	1.8 × 10^−9^	0.34	−3.89	1.0 × 10^−4^
Identified in MTAG	rs4557006	2	22443840	A	G	0.45	4.26	2.0 × 10^−5^	5.83	5.7 × 10^−9^	0.45	5.53	3.2 × 10^−8^
	rs1504930	5	155852066	T	C	0.62	−4.26	2.0 × 10^−5^	−5.58	2.5 × 10^−8^	0.62	−4.20	2.7 × 10^−5^
	rs8059002	16	25417390	T	G	0.86	−4.43	9.3 × 10^−6^	−5.46	4.8 × 10^−8^	0.85	−1.50	.13
	rs7264419	20	47701309	A	G	0.75	−5.06	4.1 × 10^−7^	−5.85	5.0 × 10^−9^	0.76	0.55	.58

A1, allele 1 (coded); Freq, frequency; A2, allele 2; Chr, chromosome; GWAS, genome-wide association study; MTAG, Multi-Trait Analysis of GWAS; MVP, Million Veteran Program; MVP_TOT_, MVP total PTSD symptoms; PGC-PTSD, Psychiatric Genomics Consortium–posttraumatic stress disorder; rsID, reference SNP ID number.

a Base pair position on chromosome (hg19/GR37 Human Genome Build).

b Significant in MVP if *p* < .006 (Bonferroni-corrected for 9 loci).

c Linkage disequilibrium proxy for rs2721817, the leading single nucleotide polymorphism in this locus.

**Table 2.** Genome-wide Significant Loci From GWASs of LTE

rsID	Chr	Position^a^	A1	A2	A1 Frequency	*z* Score	*p* Value
rs6661135	1	150999414	C	T	0.93	−5.52	3.3 × 10^−8^
rs4665501	2	22546151	G	T	0.44	−5.77	7.7 × 10^−9^
rs4704792	5	155757946	A	T	0.26	5.75	9.2 × 10^−9^
rs1476535	7	114071035	C	T	0.44	−5.77	8.0 × 10^−9^
rs2933196	14	47285917	G	A	0.59	−5.51	3.6 × 10^−8^
rs770444611	19	46917381	INS^b^	T	0.59	5.66	1.5 × 10^−8^

A1, allele 1 (coded); A2, allele 2; Chr, chromosome; GWAS, genome-wide association study; LTE, lifetime trauma exposure; rsID, reference SNP ID number.

a Base pair position on chromosome (hg19/GR37 Human Genome Build).

b Insertion of TGAGGCCAGGAGTTC.

**Table:** KEY RESOURCES TABLE

Add additional rows as needed for each resource type	Include species and sex when applicable.	Include name of manufacturer, company, repository, individual, or research lab. Include PMID or DOI for references; use “this paper” if new.	Include catalog numbers, stock numbers, database IDs or accession numbers, and/or RRIDs. RRIDs are highly encouraged; search for RRIDs at https://scicrunch.org/resources.	Include any additional information or notes if necessary.
Deposited Data; Public Database	Million Veteran Program Summary Data	PMID: **33510476**	NA	dbGaP Study Accession phs001672.v4.p1
Deposited Data; Public Database	PGC-PTSD Genotype and Phenotype data	PMID: **31594949**	NA	https://www.med.unc.edu/pgc/shared-methods/data-access-portal/
Deposited Data; Public Database	UK BioBank	PMID: **30305743**	NA	https://www.ukbiobank.ac.uk/
Software; Algorithm	FUMA version 1.3.6a	PMID:29184056	**RRID:SCR_017521**	https://fuma.ctglab.nl/
Software; Algorithm	METAL	PMID: **20616382**	**RRID:SCR_002013**	http://csg.sph.umich.edu//abecasis/Metal/
Software; Algorithm	Bolt LMM	PMID: 25642633	NA	https://www.hsph.harvard.edu/po-ru-loh/software/
Software; Algorithm	Ricopili Genetic Pipeline	PMID: **31393554**	**RRID:SCR_004496**	http://www.broadinstitute.org/mpg/ricopili/
Software; Algorithm	LD Score Regression	PMID: **25642630**	NA	https://github.com/bulik/ldsc
Software; Algorithm	LD Hub	PMID: 27663502	NA	http://ldsc.broadinstitute.org
Software; Algorithm	GWAS Atlas	PMID: **31566222**	NA	https://atlas.ctglab.nl/
Software; Algorithm	FastQTL	PMID: **26708335**	**RRID:SCR_016093**	http://fastqtl.sourceforge.net/
Software; Algorithm	MTAG	PMID: **29292387**	NA	https://github.com/JonJala/mtag
