# Supplementary material for: Enhancing Discovery of Genetic Variants for Posttraumatic Stress Disorder Through Integration of Quantitative Phenotypes and Trauma Exposure Information
Source: Biol Psychiatry. Author manuscript; Available in PMC 2022 Apr 1. (PMC8917986; doi:10.1016/j.biopsych.2021.09.020)
Supplement: mmc3 [file NIHMS1763555-supplement-mmc3.pdf]

Enhancing Discovery of Genetic Variants for Posttraumatic Stress Disorder Through Integration of Quantitative Phenotypes and Trauma Exposure Information

Supplement 1

Contents

Construction of a LTE count in the UKBB 2

Parameters for FUMA analysis 2

MTAG maxFDR statistic 5

eQTL analyses 5

PheWAS 6

Study specific acknowledgements 7

Supplementary figures 16

Construction of a LTE count in the UKBB

Participating cohorts typically provided trauma exposure as a count variable for the number of traumas. This variable did not exist in the UKBB data, which had item level data but no sum score. We constructed a count measure of LTE from 8 trauma items of the self-reported retrospective trauma screener from the UKBB mental-health questionnaire, including exposure to combat or war zone (UKBB Item: 20527), life threatening accident (UKBB Item: 20526), life threatening illness (UKBB Item: 20528), physical violence by partner or ex-partner as an adult (UKBB Item: 20523), victim of sexual assault (UKBB Item: 20531), sexual interference by partner as an adult (UKBB Item: 20524), victim of violent crime (UKBB Item: 20529), and witnessed death (UKBB Item: 20530). For our measure of LTE, items were re-coded based on the presence or absence of trauma, where 'Prefer not to answer' was coded as NA, 'never' was coded as 0, and any amount of endorsement of trauma was coded as 1. The LTE score was taken as the sum of these 8 dichotomously coded items, yielding a score of 0-8. As PTSD prevalence may be conditional on trauma type, as a sensitivity analysis, we also created a LTE phenotype where items were weighted by the trauma specific conditional prevalences of PTSD given in Yehuda et al. and analyzed it in the same manner.

Parameters for FUMA analysis

created_at	10/30/2020 19:43
FUMA	v1.3.6a
MAGMA	v1.08
GWAScatalog	e96_r2019-09-24
ANNOVAR	2017-07-17
gwasfile	eur_PTSD_Continuous_m0_pcs_alldata_may8_2020_ukbb_trauma_trait_1.txt.gz
chrcol	CHR
poscol	BP
rsIDcol	SNP
pcol	mtag_pval
eacol	A1
neacol	A2
orcol	NA
becol	mtag_beta
secol	mtag_se
leadSNPsfile	NA
addleadSNPs	1
regionsfile	NA
N	NA

Ncol	N
exMHC	1
MHCopt	annot
extMHC	NA
ensembl	v92
genetype	protein_coding
leadP	5.00E-08
gwasP	0.05
r2	0.6
r2_2	0.1
refpanel	1KG/Phase3
pop	EUR
MAF	0
refSNPs	1
mergeDist	250
magma	1
magma_window	0
magma_exp	GTEx/v8/gtex_v8_ts_avg_log2TPM, GTEx/v8/gtex_v8_ts_general_avg_log2TPM
posMap	1
posMapWindowSize	10
posMapAnnot	NA
posMapCADDth	0
posMapRDBth	NA
posMapChr15	NA
posMapChr15Max	NA
posMapChr15Meth	NA
posMapAnnoDs	NA
posMapAnnoMeth	NA
eqtlMap	1
eqtlMaptss	PsychENCODE/PsychENCODE_eQTLs.txt.gz, CMC/CMC_SVA_cis.txt.gz, CMC/CMC_SVA_trans.txt.gz, CMC/CMC_NoSVA_cis.txt.gz, CMC/CMC_NoSVA_trans.txt.gz, BRAINEAC/CRBL.txt.gz, BRAINEAC/FCTX.txt.gz, BRAINEAC/HIPP.txt.gz, BRAINEAC/MEDU.txt.gz, BRAINEAC/OCTX.txt.gz, BRAINEAC/PUTM.txt.gz, BRAINEAC/SNIG.txt.gz, BRAINEAC/TCTX.txt.gz, BRAINEAC/THAL.txt.gz, BRAINEAC/WHMT.txt.gz, BRAINEAC/aveALL.txt.gz, GTEx/v8/Brain_Amygdala.txt.gz, GTEx/v8/Brain_Anterior_cingulate_cortex_BA24.txt.gz, GTEx/v8/Brain_Caudate_basal_ganglia.txt.gz, GTEx/v8/Brain_Cerebellar_Hemisphere.txt.gz, GTEx/v8/Brain_Cerebellum.txt.gz, GTEx/v8/Brain_Cortex.txt.gz, GTEx/v8/Brain_Frontal_Cortex_BA9.txt.gz, GTEx/v8/Brain_Hippocampus.txt.gz, GTEx/v8/Brain_Hypothalamus.txt.gz, GTEx/v8/Brain_Nucleus_accumbens_basal_ganglia.txt.gz, GTEx/v8/Brain_Putamen_basal_ganglia.txt.gz,

	GTEx/v8/Brain_Spinal_cord_cervical_c-1.txt.gz, GTEx/v8/Brain_Substantia_nigra.txt.gz
eqtlMapSig	1
eqtlMapP	1
eqtlMapCADDth	0
eqtlMapRDBth	NA
eqtlMapChr15	NA
eqtlMapChr15Max	NA
eqtlMapChr15Meth	NA
eqtlMapAnnoDs	NA
eqtlMapAnnoMeth	NA
ciMap	0
ciMapBuiltin	NA
ciMapFileN	0
ciMapFiles	NA
ciMapFDR	NA
ciMapPromWindow	NA
ciMapRoadmap	NA
ciMapEnhFilt	0
ciMapPromFilt	0
ciMapCADDth	0
ciMapRDBth	NA
ciMapChr15	NA
ciMapChr15Max	NA
ciMapChr15Meth	NA
ciMapAnnoDs	NA
ciMapAnnoMeth	NA

MTAG maxFDR statistic

Violation of a primary assumption of the MTAG model, i.e. that the variance-covariance matrix of effects is identical across SNPs, can lead to an inflated false discovery rate. MTAG simulates false discovery rate under a worst case scenario to provide an upper bound of how severely deviations will affect FDR. The max FDR statistic generated by MTAG was 0.0157, indicating that any violations from MTAG model assumptions would still result in an acceptable (commonly used acceptable false discovery rates are 5%) false discovery rate.

eQTL analyses

Considering the nine and the six GWS variants associated with PTSD (five from GWAS and 4 from MTAG; Table 1) and LTE, we tested their effect on transcriptomic regulation of the surrounding genes (± 1 Mb of the gene transcription starting site) leveraging GTEx v8 resources. Applying a genome-wide false discovery rate correction, we observed that seven PTSD-associated and four LT-trauma associated loci were related to multiple tissue-specific expression quantitative trait loci (eQTL; Supplementary Table S8).

Among the PTSD-associated loci, a widespread transcriptomic regulatory effect was observed for rs146918648 (33 gene-tissue combinations) and rs7264419 (38 gene-tissue combinations). A pervasive cross-tissue effect was observed between rs146918648 and *ZNF603P* where FDR-significant transcriptomic effect was across 16 tissues and in the multi-tissue analysis a posterior probability >90% was observed with respect to 40 out the 42 tested (multi-tissue eQTL p = 1.97e-96). Rs7264419 showed a similar cross-tissue transcriptomic regulation with respect to *ARFGEF2* (10 FDR-significant tissues; multi-tissue eQTL p = 8.66e-86) and *CSE1L* (18 FDR-significant tissues; multi-tissue eQTL p = 1.03e-138). Although transcriptomic regulation is present across PTSD-associated loci, we observed only a limited number (11%) of FDR-significant eQTLs in tissues that are expected to be relevant in PTSD pathogenesis (i.e., brain regions, 7 gene-tissue combinations, top-result *DFNA5*-rs2721816 p=2.50E-06; pituitary gland, 2 gene-tissue combinations, top-result *FOXP2*-rs10266297 p=5E-06; adrenal gland, 2 gene-tissue combinations, top-result *SGCD*-rs6896669 p=5.50E-10). Similar trend was also present with respect to splicing QTLs (sQTL; Supplementary Table S9) where only 5 out 76 gene-tissue combinations (~7%) were related to tissues expected to be involved in PTSD pathogenesis. Four of these sQTL were related to the cross-tissue effect of rs7264419 across CSE1L (10 FDR significant tissues), STAU1 (22 FDR significant tissues), ZFAS1 (25 FDR significant tissues), and ZNFX1 (2 FDR significant tissues).

Among LT-associated loci, Rs6661135 was associated with the transcriptomic regulation of 15 genes across 19 different tissues. Among them, rs6661135 regulates HORMAD1 gene expression with concordant effect across eight tissues (multi-tissue eQTL p = 1.48e-69). A similar cross-tissue effect was observed with respect to PNMAL1-rs770444611 eQTL concordant and significant across 26 tissues (multi-tissue eQTL p=4.11e-191) and SGCD-rs4704792 eQTL with concordant and significant across 6 tissues (multi-tissue eQTL p=3.8e-57). Conversely, rs1476535*T allele was associated with FOXP2 transcriptomic down-regulation in the pituitary gland (normalized effect size=-0.27, p=2e-6) and upregulation in the adrenal gland (normalized effect size=0.38, p=2.5e-5). In addition to its cross-tissue eQTL effect, rs6661135 was associated with sQTL related to three surrounding genes (Supplemental Table S9): *CDC42SE1* (p<1.8e-6), *GOLPH3L* (p<1.3e-7), and *SEMA6C* (p<3.3e-6).

PheWAS

To understand further how significant loci are associated with human traits and diseases, we conducted a PheWAS of PTSD and LTE. Considering a Bonferroni multiple testing correction accounting for the number of phenotypes available (p<1.05e-5), we identified 97 phenome-wide significant (PWS) associations with respect to LTE- loci and 200 PWS associations with respect to PTSD loci (Supplementary Table S12).

Considering the PTSD PheWAS, more than half of the significant associations were related to two domains: psychiatry (34%) and metabolism (18%). Several PTSD-associated loci showed widespread pleiotropy across multiple psychiatric traits: rs10266297 (35 PWS associations, 40% psychiatric domain, top psychiatric result: risk taking p=1.27e-11), rs10821140 (37 PWS associations, 38% psychiatric domain, top psychiatric result: loneliness p=1.11e-11), rs146918648 (44 PWS associations, 48% psychiatric domain, top psychiatric result: tenseness/restlessness p=2.13e-9). Conversely, among its 58 PWS associations, rs7264419 showed an enrichment for metabolic domain (40%, top result trunk fat-free mass p=1.3e-16) over psychiatric domain (14%, top result well-being spectrum p=1.92e-8).

In the LT PheWAS, 38 phenotypic associations were related to rs1476535 and 50% of them were with psychiatric traits including sleep duration as the strongest association (p=5.8e-11). Similarly, rs4665501 showed mainly associations with psychiatric traits (9 out of 10 reaching phenome-wide significance) with strongest significance for schizophrenia-bipolar disorder meta-analysis (p=2.97e-7). Conversely, we observed that rs2933196, rs6661135, and rs4665501 were mostly associated with metabolic traits with most significant associations for body mass index (p=9.48e-15), trunk predicted mass (p=1.26e-10), and waist-hip ratio (p=1.63e-7),

respectively. The results highlight how LT loci show pleiotropy with different phenotypic domains.

Study specific acknowledgements

Army Study to Assess Risk and Resilience in Servicemembers (NSS1, NSS2, PPDS)(ST1#14, ST1#15, ST1#16)

Funding:

Army STARRS was sponsored by the Department of the Army and funded under cooperative agreement number U01MH087981 (2009-2015) with the National Institutes of Health, National Institute of Mental Health (NIH/NIMH). Subsequently, STARRS-LS was sponsored and funded by the Department of Defense (USUHS grant number HU0001-15-2-0004). The contents are solely the responsibility of the authors and do not necessarily represent the views of the Department of Health and Human Services, NIMH, the Department of the Army, or the Department of Defense.

The Army STARRS Team consists of:

Co-Principal Investigators: Robert J. Ursano, MD (Uniformed Services University of the Health Sciences) and Murray B. Stein, MD, MPH (University of California San Diego and VA San Diego Healthcare System)

Site Principal Investigators: Steven Heeringa, PhD (University of Michigan), James Wagner, PhD (University of Michigan) and Ronald C. Kessler, PhD (Harvard Medical School)

Army liaison/consultant: Kenneth Cox, MD, MPH (US Army Public Health Center)

Other team members: Pablo A. Aliaga, MS (Uniformed Services University of the Health Sciences); COL David M. Benedek, MD (Uniformed Services University of the Health Sciences); Susan Borja, PhD (NIMH); Tianxi Cai, ScD (Harvard School of Public Health); Laura Campbell-Sills, PhD (University of California San Diego); Chia-Yen Chen, ScD (Harvard Medical School); Carol S. Fullerton, PhD (Uniformed Services University of the Health Sciences); Nancy Gebler, MA (University of Michigan); Joel Gelernter, MD (Yale University); Robert K. Gifford, PhD (Uniformed Services University of the Health Sciences); Feng He, MS (University of California San Diego); Paul E. Hurwitz, MPH (Uniformed Services University of the Health Sciences); Sonia Jain, PhD (University of California San Diego); Kevin Jensen, PhD (Yale University); Kristen Jepsen, PhD (University of California San Diego); Tzu-Cheg Kao, PhD (Uniformed Services University of the Health Sciences); Lisa Lewandowski-Romps, PhD (University of Michigan); Holly Herberman Mash, PhD (Uniformed Services University of the Health Sciences); James E. McCarroll, PhD, MPH (Uniformed Services University of the Health Sciences); Adam X. Maihofer (University of California San Diego); Colter Mitchell, PhD (University of Michigan); James A. Naifeh, PhD (Uniformed Services University of the Health Sciences); Tsz Hin Hinz Ng, MPH (Uniformed Services University of the Health Sciences); Caroline M. Nievergelt, PhD (University of California San Diego); Matthew K. Nock, PhD (Harvard University); Stephan Ripke, MD (Harvard Medical School); Nancy A. Sampson, BA (Harvard Medical School); CDR Patcho

Santiago, MD, MPH (Uniformed Services University of the Health Sciences); Ronen Segman, MD (Hadassah University Hospital, Israel); Jordan W. Smoller, MD, ScD (Harvard Medical School); Xiaoying Sun, MS (University of California San Diego); Erin Ware PhD (University of Michigan); LTC Gary H. Wynn, MD (Uniformed Services University of the Health Sciences); Alan M. Zaslavsky, PhD (Harvard Medical School); and Lei Zhang, MD (Uniformed Services University of the Health Sciences).

Ash Wednesday and IVS (BRYA)(ST1#10)
This project was funded by a National Health and Medical Research Council Grant (1073041).

Bounce Back Now (BOBA)(ST1#18)
This work was supported by 1R01MH081056 (PI: Ruggiero), 1R01MH081056-S1 (PI: Amstadter), as well as K02 AA023239 (PI: Amstadter) and K01 AA025692 (PI: Sheerin).

Childhood Trauma Study (QIMR) (ST1#30)
This work was primarily supported by National Institute of Health grants to ECN (AA13446; AA011998_5978). Additional support includes grants to ACH (AA10249, AA07728, AA11998, AA13321), NGM (AA13326), PAFM (DA12854; DA027995).

Child Trauma and Neural Systems Underlying Emotion Regulation (KMCT)(ST1#19)
This work was funded by the R01-MH103291 (PI: McLaughlin), R01-MH103291-S1 (PI: McLaughlin), and R01-MH103291-S2 (PI: McLaughlin).

Cohen Veterans Center Study and Fort Campbell study (COM1, FTCB)(ST1#50, ST1#52)
These studies were supported by the Steve and Alexandra Cohen Foundation and the Department of Defense (DoD: W81XWH-09-2-0044 to C.R.M. and W911NF-09-1-0298 to R.Y.).

Cortical Excitability: Biomarker and Endophenotype in Combat Related PTSD (WANG)(ST1#59)
This work was supported by VA Merit Review awardS 1I21RX001725-01, 1I01CX000487-01A1, and 1R34MH078854-01.

Danish military study (DAMI)(ST1#28)
This research has been conducted using the Danish National Biobank resource, supported by the Novo Nordisk Foundation. The study was supported by the Research and Knowledge Centre, The Danish Veteran Centre and funded by the Danish Ministry of Defence as part of the 3rd September 2014 agreement on strengthened initiatives for Danish veterans.

Danish iPSYCH PTSD samples (DAIP)(ST1#29)
The iPSYCH team acknowledges funding by the Lundbeck Foundation (grant numbers R102-A9118 and R155-2014- 1724) and the universities and university hospitals of Aarhus and

Copenhagen. The Danish National Biobank resource was supported by the Novo Nordisk Foundation. Data handling and analysis on the GenomeDK HPC facility was supported by NIMH (1U01MH109514-01 to Michael O'Donovan and ADB). High-performance computer capacity for handling and statistical analysis of iPSYCH data on the GenomeDK HPC facility was provided by the Centre for Integrative Sequencing, iSEQ, Aarhus University, Denmark (grant to ADB).

DCS Rothbaum Study (DCSR)(ST1#38)
For support, this study was funded by by NIMH grant R01 MH-70880 to Dr. Rothbaum, Clinicaltrials.gov identifier: NCT00356278**.**

Defining Essential Features of Neural Damage (DEFE)(ST1#12)
Congressionally Directed Medical Research Programs, W81XWH-08–2–0038
Department of Veteran Affairs Rehabilitation Research and Development Service, 1IK1RX002325, I01RX000622

Detroit Neighborhood Health Study (DNHS, ADNH)(ST1#4, ST1#45)
DNHS was funded by NIH Awards R01DA022720, R01DA022720-S1, and RC1MH088283 to Allison E. Aiello; and R01MD011728 to Monica Uddin. We are grateful to all of the participants and staff for their contributions to the DNHS.

Drakenstein Child Health Study - South African sample (SAFR)(ST1#3)
Research reported in this publication was supported by the South African Medical Research Council (SAMRC) Unit on Risk & Resilience in Mental Disorders and a Self-Initiated Research Grant (NK). The views and opinions expressed are those of the authors and do not necessarily represent the official views of the SAMRC.

EA CRASH (EACR)(ST1#42)
EA CRASH was supported by the National Institute of Arthritis and Musculoskeletal and Skin Diseases of the National Institutes of Health under Award Number R01-AR056328. The content is solely the responsibility of the authors and does not necessarily represent the views of this funding agency.

Family Study of Cocaine Dependence and Collaborative Genetic Study of Nicotine Dependence (FSCD, COGA, COGB)(ST1#7, ST1#8, ST1#9)
The Collaborative Genetic Study of Nicotine Dependence (COGEND) was supported by National Cancer Institute grant P01CA089392 to Laura Bierut. The Family Study of Cocaine Dependence (FSCD) was supported by National Institute on Drug Abuse grants R01DA013423 and R01DA019963 to Laura Bierut. Funding support for genotyping was provided by the NIH GEI (U01HG004438), the National Institute on Alcohol Abuse and Alcoholism, the National Institute

on Drug Abuse, and the NIH contract "High throughput genotyping for studying the genetic contributions to human disease" (HHSN268200782096C).

GMRF-QUT (GMFR)(ST1#55)

The Queensland Branch of the Returned and Services League of Australia (RSL) funded the PTSD Initiative at the Gallipoli Medical Research Institute. The Australian Government Department of Veterans' Affairs provided transport for eligible participants. We gratefully acknowledge the dedicated efforts of the participants and their families, and the clinical and support staff involved in data collection. Specifically, we would like to thank the members of the PTSD Initiative team: Sarah McLeay, PhD, Wendy Harvey, MPH, Madeline Romaniuk, DPsych(Clin), Darrell Crawford, MD, David Colquhoun, MBBS, Ross McD Young, PhD, Miriam Dwyer, BSc, John Gibson, MBBS, Robyn O'Sullivan, MBBS, Graham Cooksley, MBBS, Christopher Strakosch, MD, Rachel Thomson, PhD, Joanne Voisey, PhD, Bruce Lawford, MBBS. We would also like to thank Emile Touma and Vikram Goel for performing psychiatric assessments, Terence Harvey for developing the study database and QUT for financial support.

Grady Trauma Project (EGHS, GTPC)(ST1#44, ST1#47)

This study was supported by the National Institutes of Health, MH071537 and MH096764.

Injury and Traumatic Stress Consortium (INTR)(ST1#27)

The PTSD and TBI INjury and TRaUmatic STress Clinical Consortium (INTRuST) was funded by a grant from the United States Department of Defense (PI: Stein, MB): W81XWH08-2-0159. Members of the INTRuST Consortium Biorepository Working Group who contributed to this work include: Gerald A. Grant MD, Christine E. Marx MD, Mark S. George MD, Thomas W. McAllister MD, Norberto Andaluz MD, Lori Shutter MD, Raul Comibra MD, Ross D. Zafonte DO, Sonia Jain PhD, Xue-Jun Qin, and Michael Hauser PhD.

Marine Resiliency Study (MRSC, BAKE)(ST1#1,ST1#57)

The Marine Corps, Navy Bureau of Medicine and Surgery (BUMED) and VA Health Research and Development (HSR&D) provided funding for MRS data collection and analysis (PI DGB) and NIH R01MH093500 funded the GWAS assays and analysis (PI CMN). Acknowledged are Victoria B. Risbrough Ph.D (VA San Diego Healthcare System & UCSD), Mark A. Geyer (UCSD), Daniel T. O'Connor (UCSD), all MRS investigators, as well as the MRS administrative core and data collection staff listed in the Methods article (Baker et al, Prev Chronic Dis. 2012;9(10):E97). The authors also thank the Marine and Navy Corpsmen volunteers for their military service and participation in MRS.

McLean Trauma Sample (TEIC)(ST1#39)

The Kaufman lab would like to thank the participants for making this research possible; the staff of the Hill Center for Women and Proctor House II, McLean Hospital; The work was supported

by National Institute of Mental Health (NIMH) grant R21MH112956 to MLK, and NIMH fellowship grant F32MH109274 to LAML, the Anonymous Women's Health Fund to MLK, the O'Keefe Family Foundation to MLK, the Trauma Scholars Fund to MLK, and the Frazier Foundation Grant for Mood and Anxiety Research to KJR.
The Teicher Lab would like to thank all the participants for being a part of these studies and the staff of Developmental Biopsychiatry Research Program, McLean Hospital. The studies were supported by National Institute of Mental Health (NIMH) grant RO1 MH91391 and National Institute on Drug Abuse R01 DA17846 to MHT.

Mid-Atlantic Mental Illness Research Education and Clinical Center the study of Post-Deployment Mental Health Study (MIRE)(ST1#26)

Preparation of this manuscript was supported by a Clinical Sciences Research and Development (CSR&D) Research Career Scientist Award (#11S-RCS-009) to Dr. Beckham, a CSR&D Career Development Award (#IK2 CX000525) to Dr. Kimbrel, and Biomedical and Laboratory Research and Development (BLR&D) Merit Award to Dr. Beckham from the U.S. Department of Veterans Affairs (VA). This work was also supported by the VA Mid-Atlantic Mental Illness Research, Education and Clinical Center (MIRECC), the Durham Veterans Affairs Medical Center, the VA Office of Mental Health Services, and the VA Office of Research and Development. The Mid-Atlantic MIRECC Workgroup contributors for this paper include: Mira Brancu, PhD, Patrick S. Calhoun, PhD, Eric Dedert, PhD, Eric B. Elbogen, PhD, John A. Fairbank, PhD, Robin A. Hurley, MD, Jason D. Kilts, PhD, Angela Kirby, MS, Christine E. Marx, MD, MS, Scott D. McDonald, PhD, Scott D. Moore, MD, PhD, Rajendra A. Morey, MD, MS, Jennifer C. Naylor, PhD, Treven C. Pickett, PsyD, Jared Rowland, PhD, Cindy Swinkels, PhD, Steven T. Szabo, MD, PhD, Katherine H. Taber, PhD., Larry A. Tupler, PhD, Elizabeth E. Van Voorhees, PhD, H. Ryan Wagner, Ph.D., Ruth E. Yoash-Gantz, PsyD. Dedert is funded by a Department of Veterans Affairs Clinical Science Research and Development Career Development Award (IK2CX000718). Naylor is funded by a Department of Veterans Affairs Rehabilitation Research and Development Career Development Award (1lK2RX000908). Van Voorhees is funded by a Department of Veterans Affairs Rehabilitation Research and Development Career Development Award (1K2RX001298). The views expressed in this article are those of the authors and do not necessarily reflect the position or policy of the Department of Veterans Affairs or the United States government.

National Centre for Mental Health (NCMH)(ST1#41)

This project was supported by the National Centre for Mental Health (NCMH). NCMH is funded by Welsh Government through Health and Care Research Wales.

National Health and Resilience in Veterans Study (NHRV; ST1#13)

The National Health and Resilience in Veterans Study is supported by the U.S. Department of Veterans Affairs National Center for Posttraumatic Stress Disorder.

NIU Trauma Orcutt (NIUT)(ST1#40)
This study was supported by the Joyce Foundation (Dr Orcutt) and NIH, HD049907and MH085436.
Nurses Health Study II (NHS2, NHSY)(ST1#5)(ST1#22)
NHSII PTSD Sub-Study was funded by National Institute on Mental Health awards RO1 MH093612, MH078928 to Karestan C Koenen. The NHSII cohort is funded in part by UM1 CA176726.

Ohio National Guard (ONGA)(ST1#2)
The funding information for the Ohio Army National Guard cohort is:
Dept. of Army, Telecommunication and Advanced Technology Research Center (TATRC) Award #s W81XWH-15-1-0080 and W81XWH-10-1-0579 “Ohio Army National Guard Mental Health Initiative: Genetics of Risk and Resilience for Deployment-Related Stress Disorders”.

OPT and CHOICE (FEEN)(ST1#37)
This research is funded by the National Institute of Mental Health (NIMH; R01MH066347, R01MH066348) and the William T. Dahms, MD, Clinical Research Unit, funded under the Cleveland Clinical and Translational Science Award (UL1 RR024989).
Pfizer Inc. supplied the medication at no cost but had no input in the trial development, conduct, analysis, or interpretation. Drs. Zoellner, Roy-Byrne, Mavissakalian, and Feeny have no competing interests to disclose.

We would like to thank all participants, therapists, and psychiatrists involved in the study and acknowledge the vital contributions of study researchers and administrators in Seattle, Washington and Cleveland, Ohio. Specifically, we would like to thank the investigative team on the grants: Jason Doctor, Ph.D., Joshua McDavid, MD, Alice S. Friedman, MSN, ARNP, and Nora McNamara, MD. Afsoon Eftekhari, Ph.D., and Lisa Stines Doane, Ph.D. were integral in the implementation of this study. Edna Foa, Ph.D. and her team provided PE integrity ratings. We would like to acknowledge Susan Silva, Ph.D., Eric Youngstrom, Ph.D., Kevin King, Ph.D., and Andrew A. Cooper, Ph.D. for their statistical consultation and analyses.

Pregnancy Outcomes, Maternal and Infant Cohort Study (PROM)(ST1#23)
This research was supported by awards from the Eunice Kennedy Shriver Institute of Child Health and Human Development (R01-HD-059835 and R01 HD059827).

Readiness and Resilience in National Guard Soldiers (RING)(ST1#33)
Support for this study came from the Center for Veterans Research and Education.

Risbrough/Norman randomized controlled psychotherapy trial (VRIS)(ST1#58)
Samples and data collection were funded by VA Office of Clinical Science Research and Development (5IO1CX000756 to SBN) and the VA Office of Basic Science Research and

Development (1I01BX002558 to VR). Additional salary support was funded by the VA National Center for PTSD (Norman) and VA Center of Excellence for Stress and Mental Health (Risbrough).

Shared Roots (SHRS)(ST1#46)
The Shared Roots project is supported by the South African Medical Research Council for the Shared Roots Flagship Project, Grant no. MRC-RFA-IFSP-01-2013/SHARED ROOTS through funding received from the South African National Treasury under its Economic Competitiveness and Support Package. Its contents are solely the responsibility of the authors and do not necessarily represent the official views of the South African Medical Research Council. Additional funding was received from the South African Research Chairs Initiative of the South African Department of Science and Technology and National Research Foundation.

Southeastern Europe PTSD (SEEP)(ST1#49)
Recruitment of the SEE-PTSD cohort was funded by the DAAD.

STRONG STAR Genetic and Environmental Predictors of Combat-Related PTSD (STRO) (ST1#35)
Funding for the STRONG STAR sample collection was made possible by the U.S. Department of Defense through the U.S. Army Medical Research and Materiel Command, Congressionally Directed Medical Research Programs, Psychological Health and Traumatic Brain Injury Research Program awards W81XWH-08-02-109 (Alan Peterson) and W81XWH-08-02-0110 (Douglas Williamson), and W81XWH-08-02-0114 (Brett Litz).

Study of Aftereffects of Trauma: Understanding Response in National Guard (SATU)(ST1#11)
Congressionally Directed Medical Research Programs, W81XWH-08–2–0038
Department of Veteran Affairs Rehabilitation Research and Development Service, 1IK1RX002325, I01RX000622

Sydney Neuroimaging (BRY2)(ST1#36)
This project was funded by a National Health and Medical Research Council Grant (1073041).

UK Biobank (UKBB)(ST1#60)
This research has been conducted using the UK Biobank Resource, as an approved extension to application 16577 (Dr. Breen). This study represents independent research part funded by the National Institute for Health Research (NIHR) Biomedical Research Centre at South London and Maudsley NHS Foundation Trust and King's College London. The views expressed are those of the authors and not necessarily those of the NHS, the NIHR or the Department of Health and Social Care. High performance computing facilities were funded with capital equipment grants

from the GSTT Charity (TR130505) and Maudsley Charity (980). GB and JRIC acknowledge funding from Cohen Veterans Bioscience.

VA Boston-National Center for PTSD Study (NCPT, TRACT)(ST1#31, ST1#32)
This research was supported in part by National Institute of Mental Health Award RO1MH079806 (MWM), Department of Veterans Affairs, Clinical Science Research & Development Program Award 5I01CX000431-02 (MWM), Department of Veterans Affairs, Biomedical Laboratory Research & Development Program Award 1I01BX002150-01 (MWM), The Translational Research Center for TBI and Stress Disorders (TRACTS), A VA Traumatic Brain Injury National Network Rehabilitation Research and Development Center award B9254-C (R.E.M. and W.P.M.), Department of Veterans Affairs. This research is the result of work supported with resources and the use of facilities at the Pharmacogenomics Analysis Laboratory, Research and Development Service, Central Arkansas Veterans Healthcare System, Little Rock, Arkansas. This work was also supported by a Career Development Award to E. J. Wolf from the Department of Veterans Affairs, Clinical Sciences Research, and Development Program.

Vietnam Era Twin Study of Aging (VETS) (ST1#24)
This research was supported by National Institute on Aging R01 AG018386, AG022982, AG050595 (W.S.K.), R01 AG018384 (M.J.L.), R03 AG046413 (C.E.F), and K08 AG047903 (M.S.P), and the VA San Diego Center of Excellence for Stress and Mental Health Healthcare System. The content is the responsibility of the authors and does not necessarily represent official views of the NIA, NIH, or VA. The Cooperative Studies Program of the U.S. Department of Veterans Affairs provided financial support for development and maintenance of the Vietnam Era Twin Registry. We would also like to acknowledge the continued cooperation and participation of the members of the VET Registry and their families.

The Women and Children's Health Study (WACH)(ST1#43)
Funding: This study was primarily supported by the National Institute of Environmental Health Sciences (grant 1U01ES021497)

Yale-Penn Study (GSDC)(ST1#6)
This study was supported by National Institutes of Health Grants RC2 DA028909, R01 DA12690, R01 DA12849, R01 DA18432, R01 AA11330, and R01 AA017535 and the Veterans Affairs VISN 1 and VISN 4 Mental Illness Research, Educational, and Clinical Centers; and the VA National Center for PTSD Research.

Genotyping services for a part of our genome-wide association study were provided by the Center for Inherited Disease Research and the Yale Center for Genome Analysis. Center for Inherited Disease Research is fully funded through a Federal contract from the National Institutes of Health to The Johns Hopkins University (contract number N01-HG-65403).

Million Veteran Program (MVP) (replication cohort)

This research includes data from the Million Veteran Program (MVP), Office of Research and Development, Veterans Health Administration, and was supported by MVP and the VA Cooperative Studies Program (CSP) study #575B.

Supplementary Figures

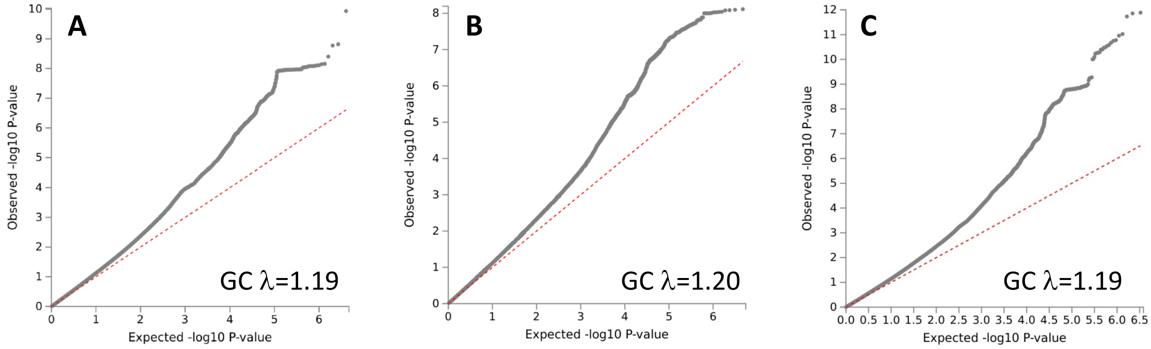

Supplementary Figure S1.

Quantile-Quantile plots. The x-axis is the expected -log10 P value under the null distribution. The y-axis is the observed -log10 p value. The dotted red line is where x = y. The grey dots denote the -log 10 P values of a given SNP. Panel A depicts the QQ plot for the PTSD GWAS. Panel B depicts the QQ plot for the LT GWAS. Panel C depicts the QQ plot for the MTAG GWAS.

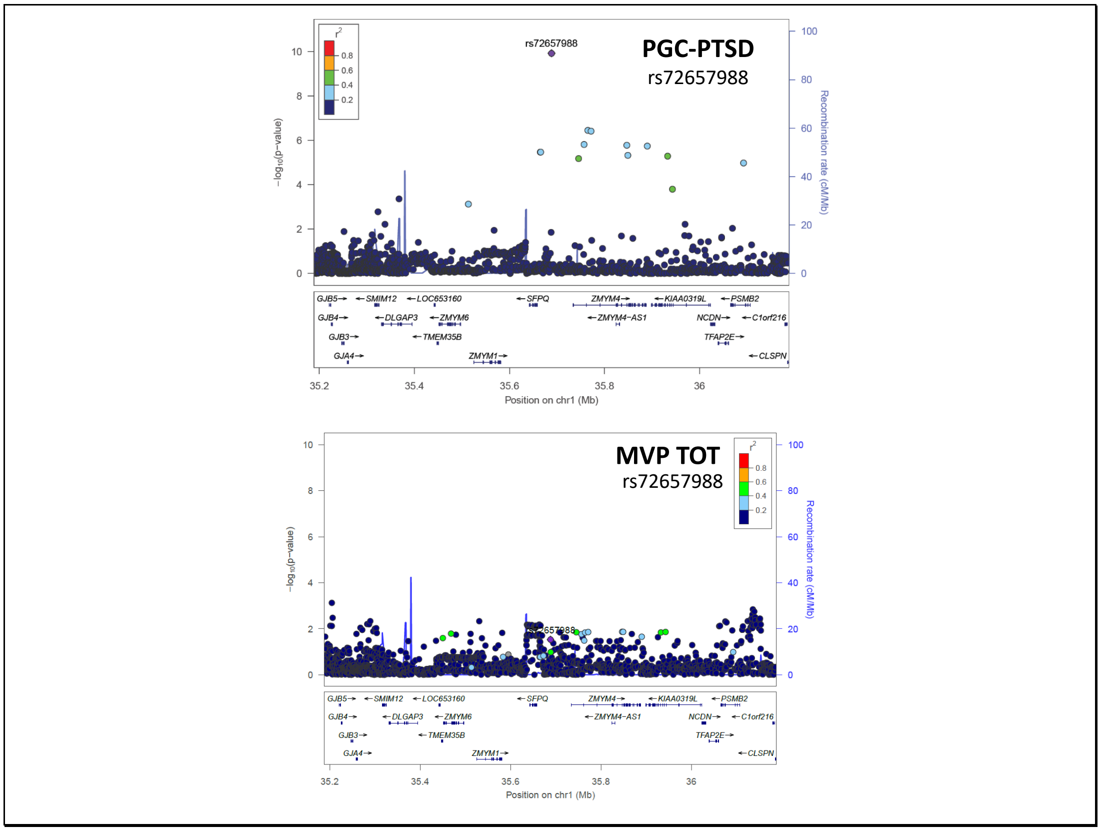

Supplementary Figure S2.

Regional plots (Locus zoom) of the top hit rs72657988. The top panel depicts the region in the PGC PTSD meta-analysis. The bottom panel depicts the region in the MVP PCL Total Severity Score cohort.

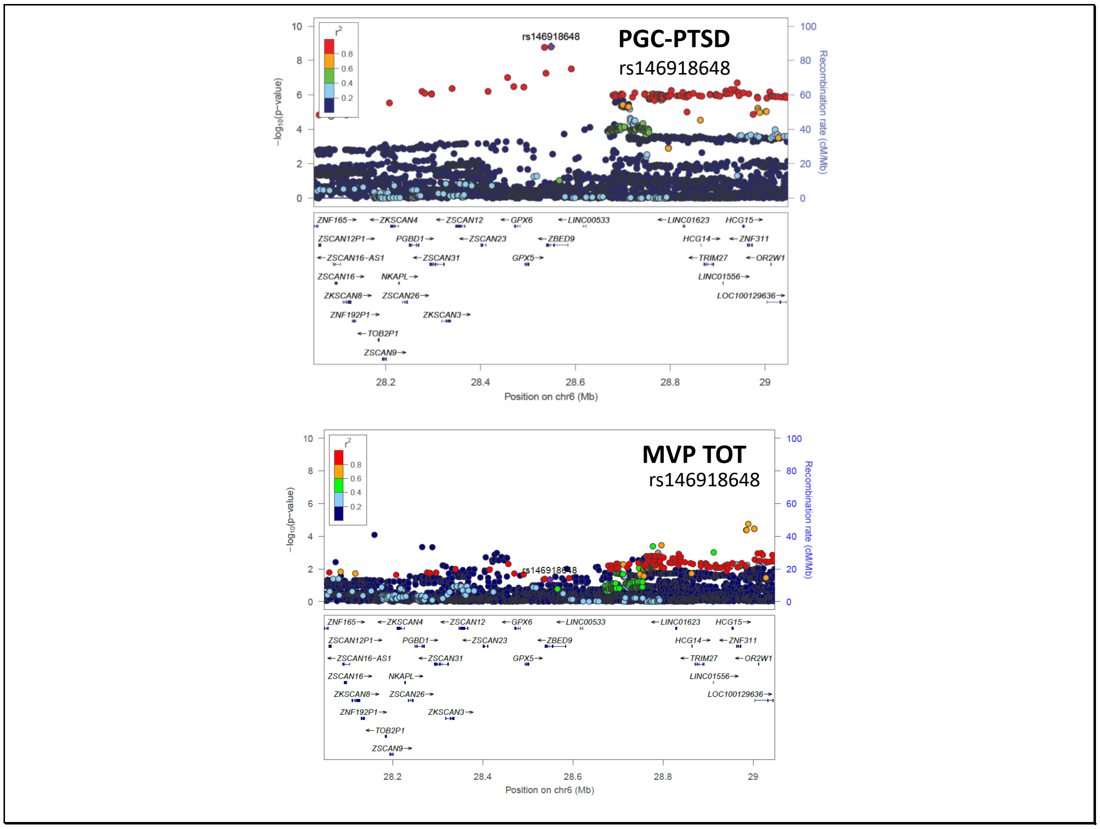

Supplementary Figure S3.

Regional plots (Locus zoom) of the top hit rs146918648. The top panel depicts the region in the PGC PTSD meta-analysis. The bottom panel depicts the region in the MVP PCL Total Severity Score cohort.

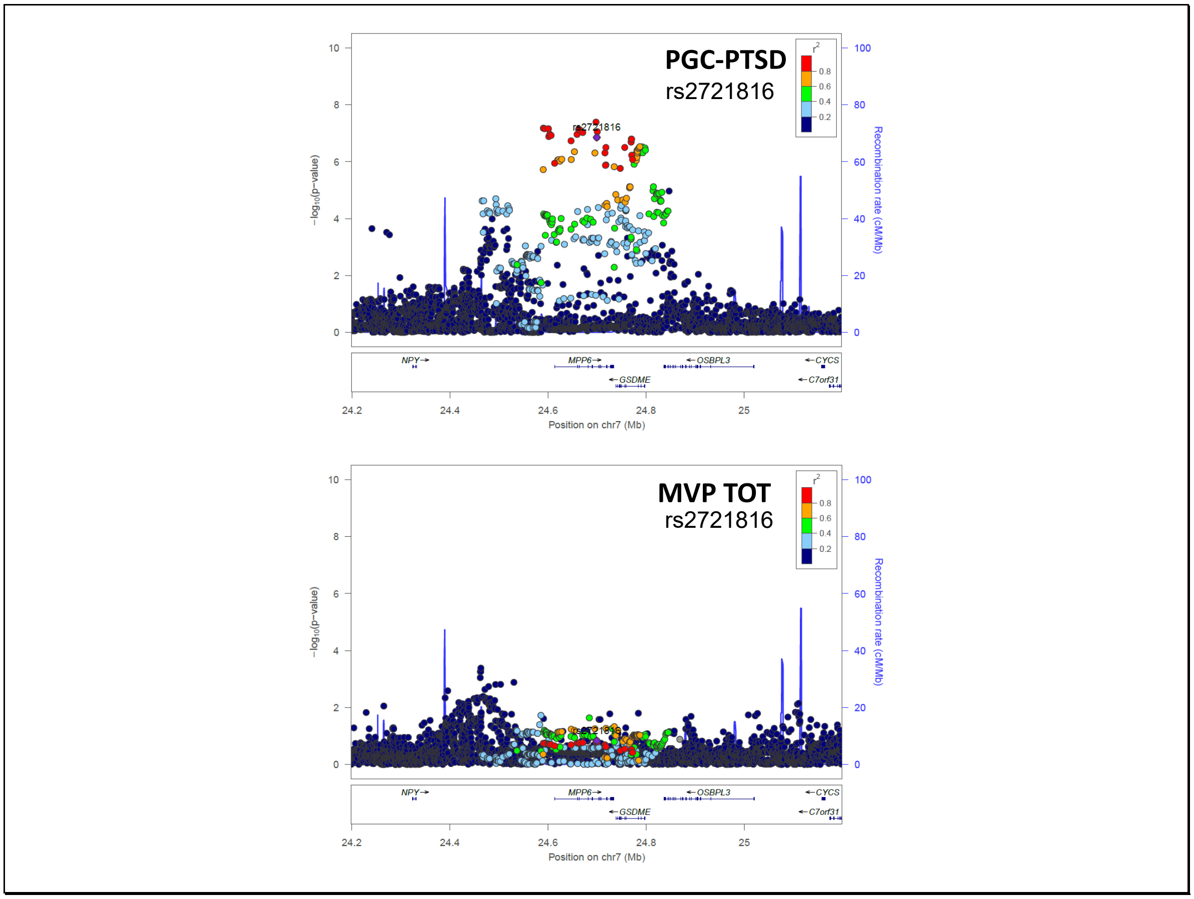

Supplementary Figure S4.

Regional plots (Locus zoom) of the top hit rs2721816. The top panel depicts the region in the PGC PTSD meta-analysis. The bottom panel depicts the region in the MVP PCL Total Severity Score cohort.

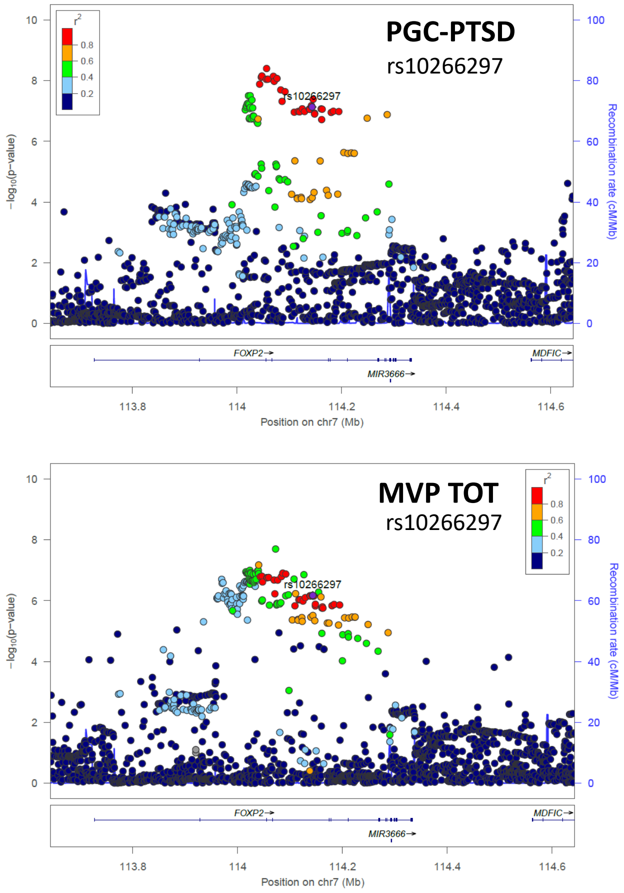

Supplementary Figure S5.

Regional plots (Locus zoom) of the top hit rs10266297. The top panel depicts the region in the PGC PTSD meta-analysis. The bottom panel depicts the region in the MVP PCL Total Severity Score cohort.

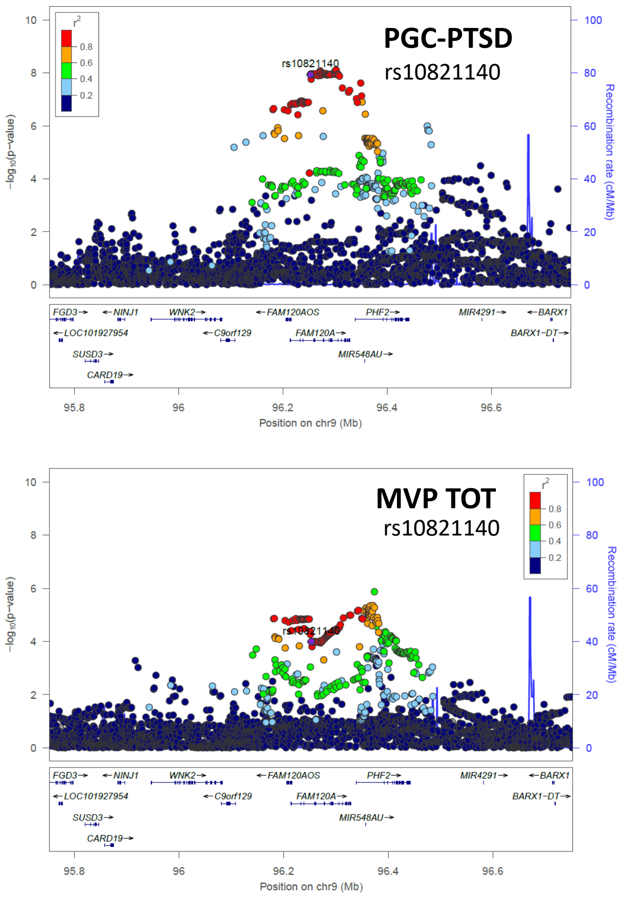

Supplementary Figure S6.

Regional plots (Locus zoom) of the top hit rs10821140. The top panel depicts the region in the PGC PTSD meta-analysis. The bottom panel depicts the region in the MVP PCL Total Severity Score cohort.

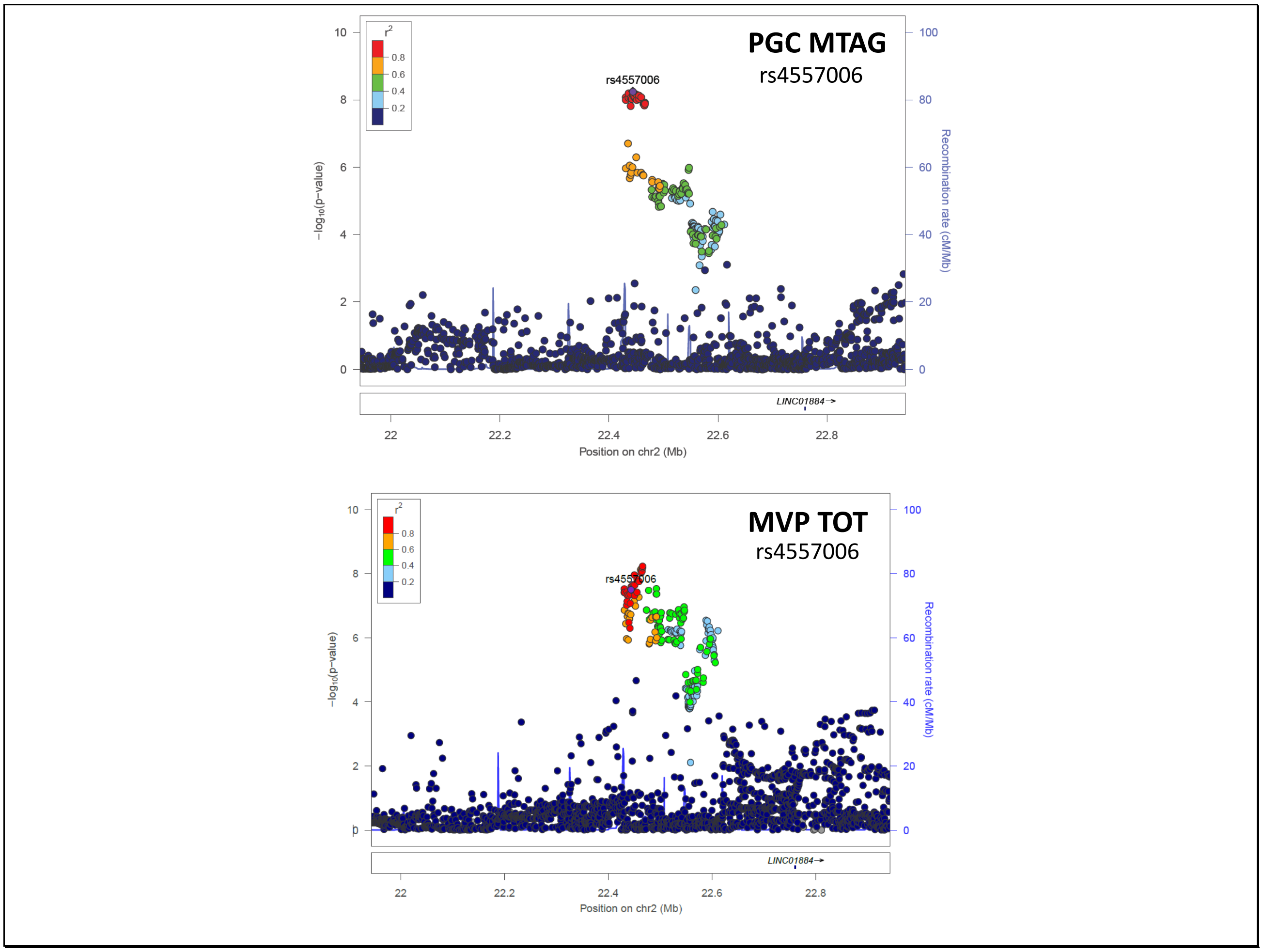

Supplementary Figure S7.

Regional plots (Locus zoom) of the top hit rs4557006. The top panel depicts the region in the PGC MTAG analysis. The bottom panel depicts the region in the MVP PCL Total Severity Score cohort.

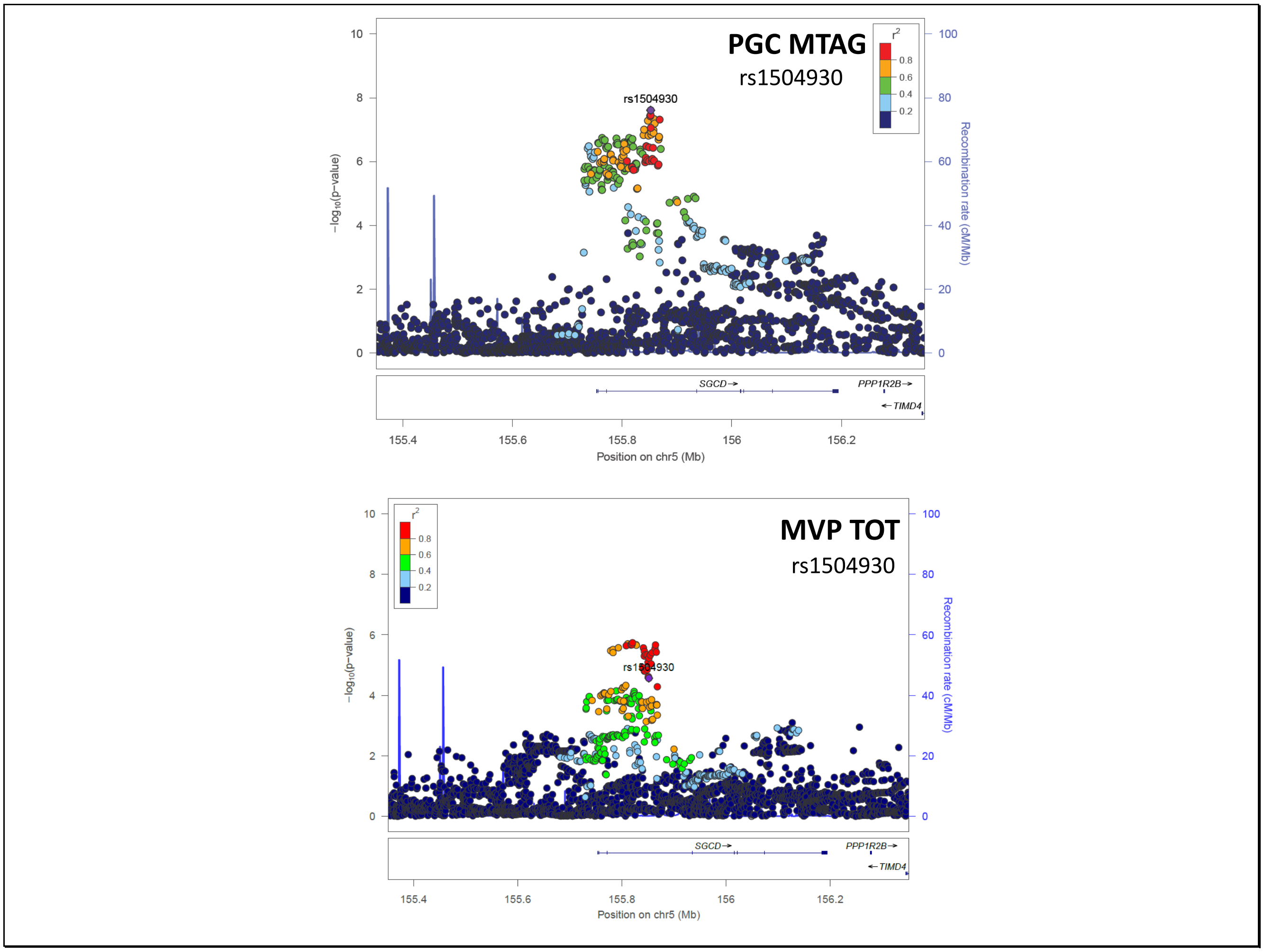

Supplementary Figure S8.

Regional plots (Locus zoom) of the top hit rs1504930. The top panel depicts the region in the PGC MTAG analysis. The bottom panel depicts the region in the MVP Re-experiencing cohort.

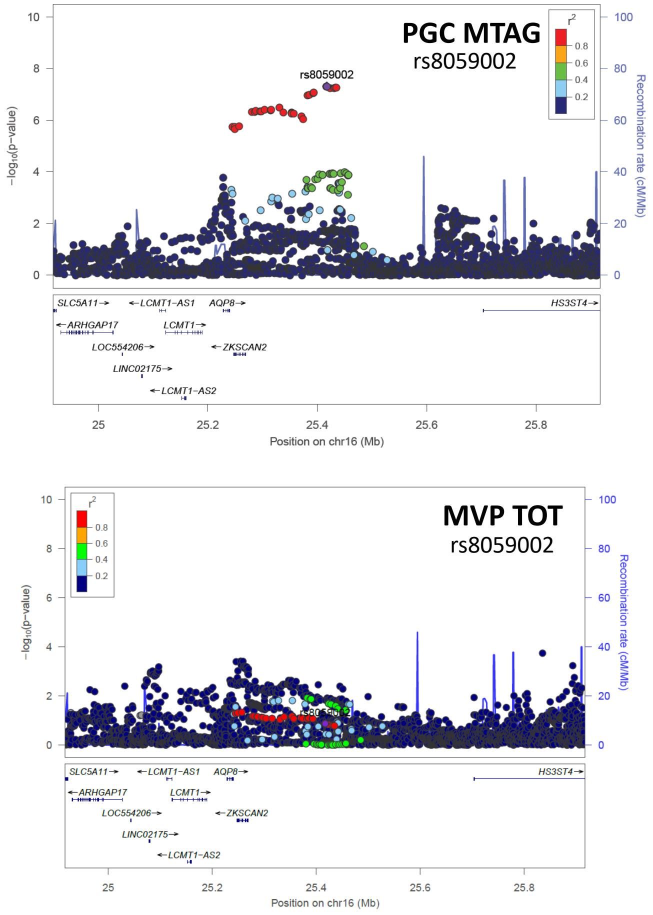

Supplementary Figure S9.

Regional plots (Locus zoom) of the top hit rs8059002. The top panel depicts the region in the PGC MTAG analysis. The bottom panel depicts the region in the MVP PCL Total Severity Score cohort.

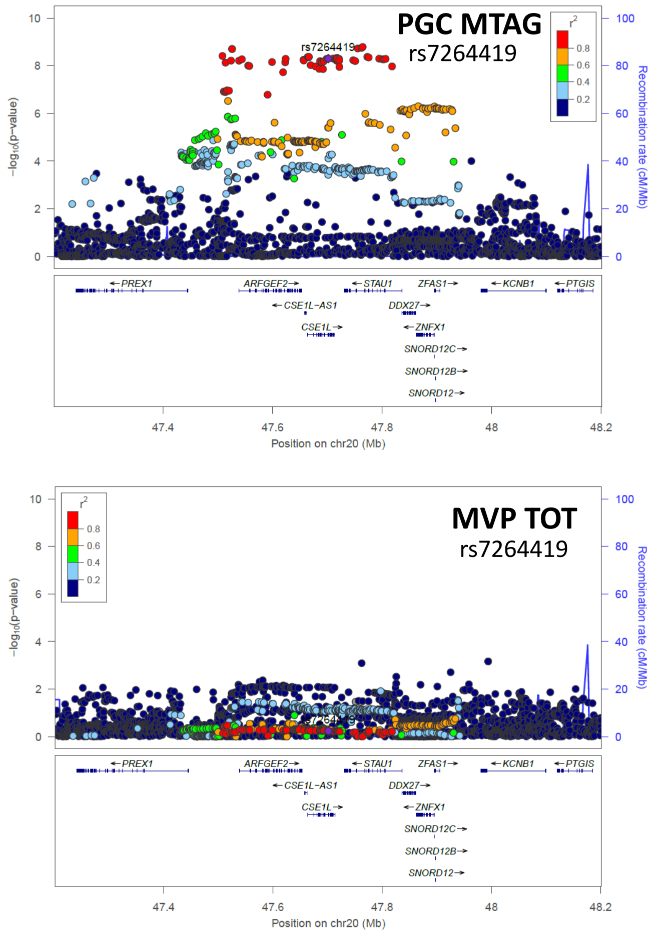

Supplementary Figure S10.

Regional plots (Locus zoom) of the top hit rs7264419. The top panel depicts the region in the PGC MTAG analysis. The bottom panel depicts the region in the MVP PCL Total Severity Score cohort.
